# Epigenomic profiling of non-small cell lung cancer xenografts uncover *LRP12* DNA methylation as predictive biomarker for carboplatin resistance

**DOI:** 10.1186/s13073-018-0562-1

**Published:** 2018-07-20

**Authors:** Sabrina Grasse, Matthias Lienhard, Steffen Frese, Martin Kerick, Anne Steinbach, Christina Grimm, Michelle Hussong, Jana Rolff, Michael Becker, Felix Dreher, Uwe Schirmer, Stefan Boerno, Anna Ramisch, Gunda Leschber, Bernd Timmermann, Christian Grohé, Heike Lüders, Martin Vingron, Iduna Fichtner, Sebastian Klein, Margarete Odenthal, Reinhard Büttner, Hans Lehrach, Holger Sültmann, Ralf Herwig, Michal R. Schweiger

**Affiliations:** 1 Translational Epigenetics and Tumor Genetics, University Hospital Cologne, Cologne, Germany; 2 Department of Computational Molecular Biology, Max Planck Institute for Molecular Genetics, Berlin, Germany; 3 ELK Berlin Chest Hospital, Berlin, Germany; 4 Department of Vertebrate Genomics, Max Planck Institute for Molecular Genetics, Berlin, Germany; 5 Present Address: Department of Cell Biology and Immunology, Institute for Parasitology and Biomedicine, Granada, Spain; 6 Department of Biology, Chemistry and Pharmacy, Free University Berlin, Berlin, Germany; 7 Center for Molecular Medicine Cologne, CMMC, Cologne, Germany; 8 Experimental Pharmacology and Oncology Berlin-Buch GmbH, Berlin, Germany; 9 Alacris Theranostics GmbH Berlin, Berlin, Germany; 10 Cancer Genome Research Group, German Cancer Research Center (DKFZ), German Cancer Consortium (DKTK), and National Center for Tumor Diseases (NCT), Heidelberg, Germany; 11 Translational Lung Research, Center (TLRC), German Center for Lung Research (DZL), Heidelberg, Germany; 12 Sequencing Core Facility, Max Planck Institute for Molecular Genetics, Berlin, Germany; 13 Institute of Pathology, University of Cologne, Cologne, Germany; 14 Else Kröner Forschungskolleg Clonal Evolution in Cancer, University Hospital Cologne, Weyertal 115b, 50931 Cologne, Germany

**Keywords:** Non-small cell lung cancer, NSCLC, Epigenomics, Predictive biomarker, Therapy response, DNA methylation, Patient-derived xenografts, Carboplatin resistance

## Abstract

**Background:**

Non-small cell lung cancer (NSCLC) is the most common cause of cancer-related deaths worldwide and is primarily treated with radiation, surgery, and platinum-based drugs like cisplatin and carboplatin. The major challenge in the treatment of NSCLC patients is intrinsic or acquired resistance to chemotherapy. Molecular markers predicting the outcome of the patients are urgently needed.

**Methods:**

Here, we employed patient-derived xenografts (PDXs) to detect predictive methylation biomarkers for platin-based therapies. We used MeDIP-Seq to generate genome-wide DNA methylation profiles of 22 PDXs, their parental primary NSCLC, and their corresponding normal tissues and complemented the data with gene expression analyses of the same tissues. Candidate biomarkers were validated with quantitative methylation-specific PCRs (qMSP) in an independent cohort.

**Results:**

Comprehensive analyses revealed that differential methylation patterns are highly similar, enriched in PDXs and lung tumor-specific when comparing differences in methylation between PDXs versus primary NSCLC. We identified a set of 40 candidate regions with methylation correlated to carboplatin response and corresponding inverse gene expression pattern even before therapy. This analysis led to the identification of a promoter CpG island methylation of LDL receptor-related protein 12 (*LRP12*) associated with increased resistance to carboplatin. Validation in an independent patient cohort (*n* = 35) confirmed that *LRP12* methylation status is predictive for therapeutic response of NSCLC patients to platin therapy with a sensitivity of 80% and a specificity of 84% (*p* < 0.01). Similarly, we find a shorter survival time for patients with *LRP12* hypermethylation in the TCGA data set for NSCLC (lung adenocarcinoma).

**Conclusions:**

Using an epigenome-wide sequencing approach, we find differential methylation patterns from primary lung cancer and PDX-derived cancers to be very similar, albeit with a lower degree of differential methylation in primary tumors. We identify *LRP12* DNA methylation as a powerful predictive marker for carboplatin resistance. These findings outline a platform for the identification of epigenetic therapy resistance biomarkers based on PDX NSCLC models.

**Electronic supplementary material:**

The online version of this article (10.1186/s13073-018-0562-1) contains supplementary material, which is available to authorized users.

## Background

Lung cancer is the leading cause of cancer-related deaths worldwide. Non-small cell lung cancer (NSCLC) comprises approximately 85% of all lung cancers [1]. Besides radiation and surgery, NSCLC is treated with chemotherapeutic agents like platinum-based drugs and targeted therapies. Nonetheless, the 5-year overall survival rate of NSCLC patients is only 18% [2]. A major problem is the intrinsic or acquired therapy resistance, occurring in approximately 50% of all cases. Thus, predictive biomarkers to optimize patient’s therapy are urgently needed.

DNA methylation is one of the most stable epigenetic modifications in mammalian cells. Furthermore, aberrant DNA methylation is a feature not only of early carcinogenesis, but also of therapy resistance mechanisms which makes them promising biomarker candidates [3]. In NSCLC, systematic genome-wide approaches have identified global hypomethylations and promoter-associated hypermethylations, either by high-throughput methods or targeted approaches [4–11].

Recently, several biomarkers predicting the therapy response in lung cancer have been suggested including *IGFBP3* (insulin-like growth factor binding protein 3), *TGM2* (transglutaminase 2), and *SLFN11* (Schlafen family member 11) for cisplatin and *DAPK* (death-associated protein kinase) for erlotinib and cetuximab. All of them were identified in cell lines and encode transcription factors involved in cellular responses to stress and development [12–15]. Of these, only *IGFBP3* and *SLFN11* were additionally tested in clinical tissue samples derived from NSCLC patients but still showed a rather poor predictive power in lung cancer [15, 16]. These values are increased when *IGFBP-3* methylation values are combined with EGFR, IGFIR, and AKT protein phosphorylation [12]. However, for these analyses, additional three immunohistochemistries are needed which render the assays highly sensitive to inter-laboratory variability and are more labor-intense than sole DNA methylation analyses.

One possible explanation for the rather low comparability to clinical samples may be due to the cell line-based primary screening approach, as cancer cell lines have adapted to ex vivo growth and thus do not appropriately model primary tumors [17, 18]. For this reason, we aimed to identify therapy resistance biomarkers in patient-derived NSCLC xenograft (PDX) models. PDX models have been shown to closely mimic the primary tumor profiles of gene copy number, mutation distribution, gene expression, and phosphoproteome level [17–19]. Furthermore, studies with 450 K methylation arrays showed that PDXs are more similar to primary tumors than cell lines and that they clustered according to their pathohistological subtype [17, 18].

We analyzed genome-wide methylation patterns in PDX models derived from 22 NSCLC patients, as well as corresponding normal lung tissues. For each model, we determined the tumor response rate by treating the animals with carboplatin. Next to DNA methylation analyses, we also performed gene expression analyses and integrated all data with the respective carboplatin response. We identified *LRP12* as a resistance biomarker in the PDX models and further validated the methylation differences of responders and non-responders in an independent cohort with single-locus methylation analyses in 35 additional formalin-fixed and paraffin-embedded (FFPE) primary tumor samples. Finally, examination of clinical follow-up data from our cohort as well as from the TCGA data set revealed that patients with *LRP12* promoter hypermethylation have significantly decreased progression-free and overall survival after platinum therapy.

## Methods

### Experimental design

The objective of this study was to investigate DNA methylation alterations associated with intrinsic resistance and to identify biomarkers predicting patient’s outcome in NSCLC. We used 22 NSCLC PDXs and corresponding normal tissues to determine the therapy response to carboplatin for each tumor, generated genome-wide DNA methylation and gene expression profiles of each model, and used them to identify epigenetic therapy resistance biomarkers. Sample size ranged from *n* = 22 (PDXs and corresponding lung normal tissue) to 32 PDXs for methylation or expression analyses, respectively. Candidate biomarkers were validated in an independent human primary NSCLC cohort for which response data was obtained by clinical investigations. According to availability, material of 35 formalin-fixed embedded samples was analyzed by methylation-specific PCR (PCR) by quantitative methylation-specific PCR (qMSP).

### Clinical samples

Two independent sets of clinical samples were used: (1) patient-derived xenograft models for genome-wide methylation analysis and identification of potential epigenetic therapy resistance mechanisms and predictive biomarkers and (2) FFPE samples of primary NSCLC patients to validate selected biomarker candidates.

Establishment of PDX models from NSCLC patients was ethically approved (EA3/001/06) by the local ethical review committee (Charité, Berlin). All mice used in the study were handled in accordance with the Guidelines for the Welfare and Use of Animals in Cancer Research [20]. Their use was approved by the local responsible authorities (approval no. G 0030/15), according to the German Animal Protection Law. Patient lung tumor samples were implanted subcutaneously into 1–3 nude or NOD/SCID mice (in-house breeding) [21]. For the generation of PDXs, we used primary NSCLC tumor samples with a tumor cell content ranging from 5% to more than 70%. Commonly, cohorts are preselected for high tumor content (e.g., > 60% in The Cancer Genome Atlas (TCGA)) to facilitate analysis of tumor-specific features. However, this filtering excludes a large fraction of samples and is thus not appropriate for diagnostic purposes. Therefore, we implicitly included samples with low tumor content in our study.

For each PDX model, six mice were exposed to carboplatin (75 mg/kg/day) per injection or solvent intraperitoneal at days 1 and 8 and tumor growth was measured by caliper measurement for 2–6 weeks.

Once tumors became palpable, tumor size was measured weekly with a caliper-like instrument. Individual tumor volume *V* was calculated with the following formula: *V =* ½ length × width^2^*.* Tumors of each model were further transplanted into 2–4 mice after a tumor volume of approx. 1.2 cm^3^ was reached. Where possible, snap-frozen tumor samples from each passage (up to ten passages) were conserved and stored at − 80 °C for further analysis. Patients’ data and clinical characteristics like age, sex, tumor stage, smoking history, prior treatment, and histology of primary NSCLC used for the establishment of PDX are given in Additional file 1: Table S1. Chemosensitivity testing was performed as described before in male NMRI:nu/nu mice [22]. To this end, 6 mice were randomly assigned to each control or treatment group. Treated to control (T/C) values of relative tumor volume were used for the evaluation of the treatment.

For the validation, cohort patients who underwent radical (R0) surgery for NSCLC followed by adjuvant chemotherapy with cis-/carboplatin were selected from the cancer database of the Chest Hospital Berlin. Thirty-five pairs of patients with and without relapse of NSCLC were identified and matched for age, sex, histological subtype, and tumor stage. Tumor tissue of these patients was collected from formalin-fixed paraffin-embedded (FFPE) material. The use of clinical data and patient’s material was approved by the institutional review board of the ELK Berlin Chest Hospital.

### Methylation profiling by MeDIP-Seq

Isolation of DNA and RNA from frozen tissue samples was performed using a TissueLyser and the AllPrep DNA/RNA/Protein Mini Kit according to manufacturer’s recommendations. For MeDIP-Seq analyses, 1.3 μg of genomic DNA was randomly sheared using the Covaris S2 or M system to assess a size range of 100 to 300 bp. TruSeq DNA Sample Preparation Kit (Illumina) was used to perform Illumina library preparation. Fragmented DNA was end-repaired into dA-tailed fragments, and TruSeq indexed adaptors were ligated. Library preparation reactions were cleaned up by AMPure XP beads (Beckman Coulter). Adapter-ligated DNA was denatured at 95 °C for 10 min and subjected to the methylated DNA immunoprecipitation (MeDIP) procedure. MeDIP was performed using 5 μg of a monoclonal antibody directed against 5-methylcytidine (Eurogentec) and coupled to magnetic Dynabeads with M-280 sheep antibody to mouse IgG (Thermo Fisher Scientific). Denatured DNA and antibody coupled to the magnetic beads were incubated at 4 °C for 4 h in IP buffer (10 mM sodium phosphate buffer (pH 7.0), 140 mM NaCl, 0.25% Triton X100). The reaction was washed three times with IP buffer, and DNA was eluted from the beads in elution buffer (50 mM Tris-HCl (pH 7.5), 10 mM EDTA, 1% SDS) at 65 °C for 15 min. To separate the antibody, the DNA was treated with proteinase K for 2 h at 55 °C and methylated DNA was recovered using the QIAquick PCR Purification Kit. MeDIP efficiency was conducted with quantitative PCR (qPCR) targeting self-designed spiked-in controls as well as further methylated and unmethylated genomic regions [23]. On average, 93.5% specificity and 30.3 fold enrichment of immunoprecipitation reaction using spiked-in controls were achieved.

Following MeDIP enrichment, libraries were PCR-amplified, size-selected, and quantified using the Quant-iT dsDNA HS Assay Kit and a Qubit 1.0 Fluorometer (Invitrogen). Libraries were multiplexed and sequenced using the Illumina HiSeq2000 platform.

### Processing of MeDIP-Seq data

MeDIP paired-end reads were aligned using bwa Version 0.7.12-r1044 with default parameters [24]. In order to remove sequencing reads that originated from mouse DNA fragments, MeDIP reads from both PDX and human tissue samples were aligned to the mouse/mm10 reference sequence first. Only read pairs that did not align to the mouse reference were aligned to the human reference GrCh37/hg19 and processed in R 3.3.2 with QSEA version 1.0 [25]. According to the average fragment lengths, which are ranging from 177 to 277 bases for the individual samples, the size of the genome-wide windows was set to 250 bases. CNVs were calculated from MeDIP data by considering only fragments without any CpG, based on 1 Mb windows. CpG enrichment profiles were calibrated based on mean Illumina 450 k methylation values from TCGA LUSC and LUAD cohorts (*n* = 172) and regions with mean methylation > 90% and variance < 0.05 [26, 27]. Subsequently, region-wise methylation levels were estimated for all samples and used for PCA analysis.

DMRs between PDX and normal were detected with the QSEA software, by estimating the region-wise maximum likelihood differences of average MeDIP enrichment rate between PDX and normal using generalized linear models (GLMs). These likelihoods were compared to the maximum likelihood average rates for all samples, using likelihood ratio tests. From the test statistics, the false discovery rates (FDR) were computed, to adjust for multiple testing [28]. The same approach was applied in order to detect DMR associated with therapy response (rDMRs), using the log relative tumor volume as a quantitative predictor, on which the enrichment depends in the GLM. LHBs were defined as regions that are at least 1 Mb in size, with at least 20% reduced average methylation level (as estimated from MeDIP-Seq) in PDX compared to normal. Within QSEA, genomic regions were annotated with gene, exon, and promoter (transcription start site ± 2 kb) information from RefSeq, ENCODE TFBS, and model-based CpG islands, all obtained via the UCSC table browser.

### Targeted bisulfite sequencing with the Methyl-Seq technology

Methyl-Seq experiments were performed using the SureSelectXT Methyl-Seq Target Enrichment System by Agilent Technologies. Briefly, 3.0 μg genomic DNA was sheared to a size range between 100 and 300 bp using the Covaris S2 or M system. Libraries were prepared according to manufacturer’s recommendations. Adapter-ligated DNA was denatured and subsequently hybridized to a RNA capture library for 24 h at 65 °C. Following the capturing of the RNA-DNA hybrids using streptavidine-coated magnetic beads, DNA was separated from the beads, eluted, and bisulfite-treated using the EpiTect Bisulfite Kit. The bisulfite-converted DNA libraries were PCR-amplified and purified. A further amplification step was performed to add barcode sequences for sample pooling and sequencing analysis via the Illumina HiSeq2000 platform. The indexed DNA pool was analyzed with the 2100 Bioanalyzer High Sensitivity DNA assay (Agilent Technologies) prior to sequencing.

### Processing of Methyl-Seq data

Adapter sequences in paired-end Methyl-Seq reads were trimmed using trim_galore version 0.4.0 and then aligned using bismark v0.10.0 based on bowtie2 version 2.2.1 with default parameters [29, 30]. Corresponding to the MeDIP alignment strategy, Methyl-Seq reads were aligned to the mouse/mm10 reference first. Read pairs, which did not match the mouse genome, were aligned to the human reference GrCh37/hg19. Methylation levels were called at CpG sites covered by 20 or more reads using bismark_methylation_extractor. To compare CpG-wise methylation levels from Methyl-Seq to region-wise MeDIP methylation levels, Methyl-Seq methylation values were averaged within 250 base regions.

### Immunohistochemistry

Tissue samples were incubated in 4% formalin for 24 to 36 h and subsequently embedded in paraffin. For tissue analysis, 3–5-μm sections were cut and deparaffinized and antigen retrieval was performed using an enzymatic approach (proteinase K), or heat antigen retrieval with either citrate at pH 6.0, or EDTA at pH 9.0 for 20 min. Washing steps were performed using phosphate-buffered saline. The following primary antibodies were used: AE1/3 (CKAE1/3, Zytomed, #AE1/AE3&5 D3), Cytokeratin 5 (CK5, Zytomed, #XM26), Cytokeratin 7 (CK7, Dako, #OV-TL12/30), CD56 (Zytomed, #123C3), TTF-1 (Dako, #8G7G3/1), and p40 (Zytomed, #polyclonal).

### siRNA knockdown of *LRP12* and cell viability assays

Details can be found in Additional file 2: Additional Methods.

### Methylation-specific PCR on FFPE material

DNA from FFPE material was isolated using Maxwell® 16 FFPE Plus LEV DNA Purification Kit (Promega) for the Maxwell® 16 instrument. Subsequently, amplifiable DNA was quantified using qPCR.

Methylation-specific primer pairs were designed using MethPrimer [31]. DNA was bisulfite-converted using the EpiTect Kit. Conversion efficiency was assessed with a Calponin-PCR [32]. Quantitative methylation-specific PCR (qMSP) was performed using KAPA SYBR FAST qPCR Master Mix (Peqlab) and primers LRP12_M_fw 5′-tcgaaaggagttatttttaattcga-3′, LRP12_M_rev 5′-tacaaaatcctattaattccccga-3′, LRP12_U_fw 5′-ttgaaaggagttatttttaatttga-3′, and LRP12_U_rev 5′-tacaaaatcctattaattccccaaa-3′. Amplified region comprises CpG1 (chr8: chr8:105,600,307) and CpG2 (chr8:105,600,467) and is visualized in Additional file 2: Figure S1. EpiTect control DNA was used as controls. The derived methylation levels of the FFPE samples were normalized to the corresponding region-specific values derived from the commercial control DNA. Average methylation levels were determined of resistant and sensitive patients, separately, and significance of average methylation differences between both groups was calculated for each region using a Mann-Whitney test.

### Gene expression profiling by microarray

Gene expression profiles of 32 PDXs and 22 corresponding normal lung tissues were generated using the whole-genome microarray. Briefly, RNA was isolated with the RNase-Free DNase Set according to the manufacturer’s protocol. After quality control, genome-wide gene expression analysis was carried out using the Illumina Human-HT-12 v4 Expression BeadChip gene expression platform (DKFZ Genomics and Proteomics Core Facility (GPCF)) comprising 48,107 probes. Illumina GenomeStudio v2011.1 was used for the export of summarized probe intensities. Next, the expression data were imported into R/Bioconductor for further processing together with associated metadata. Data were background-corrected, variance-stabilized, and quantile-normalized with the lumi Bioconductor package [33, 34]. Annotations for all probes were obtained via the illuminaHumanv4.db Bioconductor package [35]. Only probes considered to be of perfect or good quality according to these annotations were kept, leaving 34,410 probes for analysis. For gene-level analyses, duplicate probes were collapsed to the probe with the highest mean expression across samples, resulting in 23,777 genes. Each PDX sample was set into relation to the median of all normal tissue samples to detect differentially expressed regions (DERs).

### Statistical analysis, pathway analyses

Statistical analysis comparing two groups was performed using unpaired, two-sided Wilcoxon rank sum test (Mann-Whitney test). For the determination of survivals in relation to *LRP12* methylation status, a total of 35 patients were screened for *LRP12* promoter methylation status and were found either hypermethylated (LRP12+, 16 patients) or non-hypermethylated (LRP12−, 19 patients). Taking into consideration patients that were censored (LRP12+, 5 out of 16; LRP12−, 14 out of 19), we generated Kaplan-Meier survival curves for overall survival (OS) and progression-free survival (PFS) using the Eureka statistics online tool with 95% confidence intervals (http://eurekastatistics.com/kaplan-meier-survival-curve-grapher/). Statistical testing of differences between the survival curves was based on a log-rank test using the R/Bioconductor package “survival” [36]. For further statistical analyses of genome-wide DNA methylation and gene expression, please see under “processing of MeDIP-Seq data or Methyl-Seq data” and “gene expression profiling by microarray.”

For pathway analysis of the 2380 promoter-associated rDMRs, Ingenuity Pathway Analysis software IPA (Ingenuity Systems®, Qiagen) was used. The score (−log *p* value) of an enriched signaling pathway is calculated using Fisher’s exact test and indicates the likelihood that a gene will be found in a network due to random chance (*p* values ≤ 0.05 were considered as significant). Ingenuity’s upstream regulator analysis in IPA is a tool that predicts upstream regulators based on the literature and compiled in the Ingenuity^®^ Knowledge Base. The activation *Z*-score is an estimate of the status of the upstream regulator using the level of gene expression of known target genes. *Z*-scores greater than 2 or smaller than − 2 can be considered as significant. The overlap *p* value is calculated using Fisher’s exact test, and as significance, *p* values < 0.01 were used.

## Results

### MeDIP-Seq reveals DNA methylation profiles of PDX models of NSCLC

**Fig. 1:**
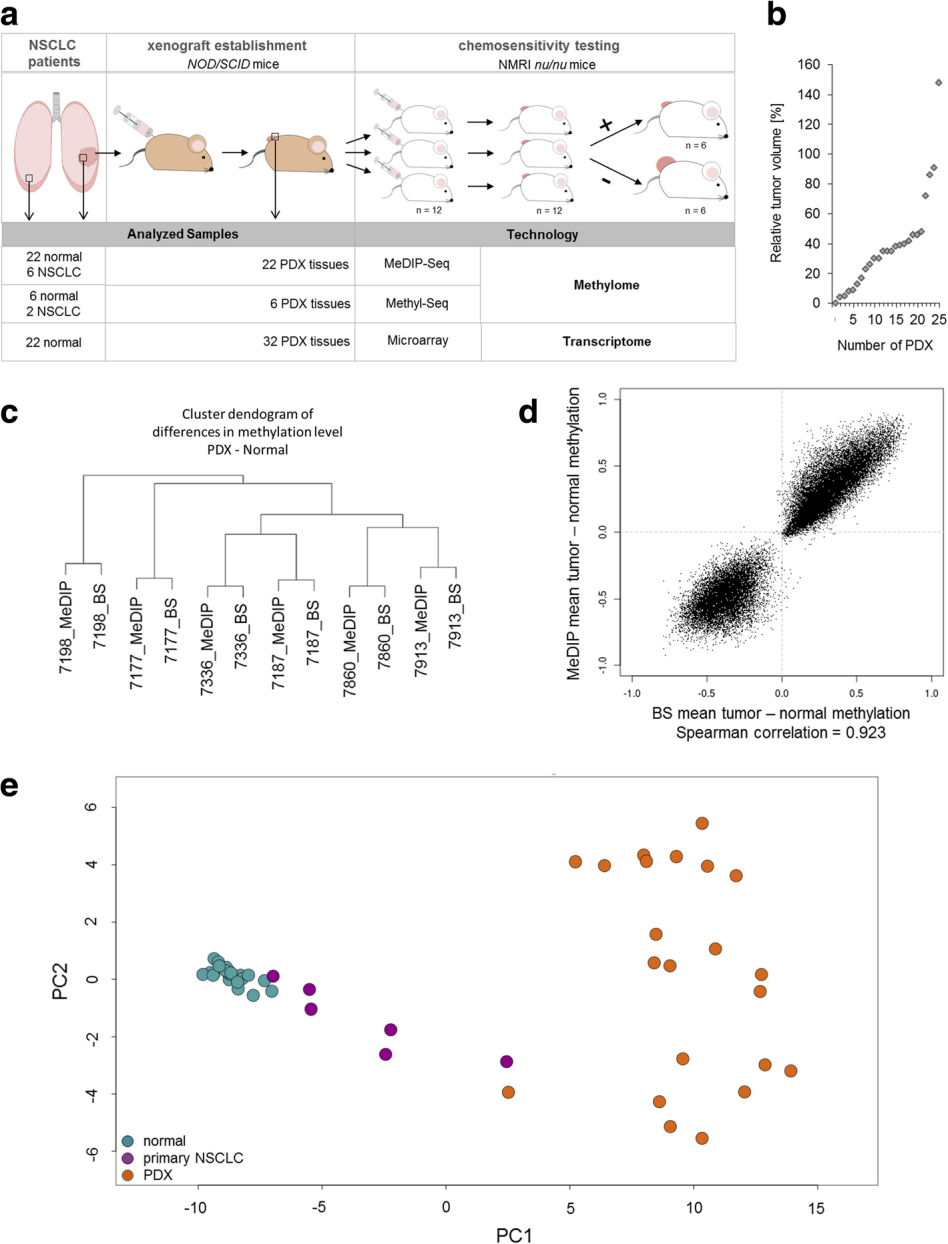
Establishment of genome-wide DNA methylation profiles of patient-derived xenograft (PDX) models of NSCLC tumors**. a** Establishment and chemosensitivity testing was performed as described before [21]. Patient’s NSCLC tumors were resected and transplanted into immunodeficient NOD/SCID mice for tumor growth. Each patient-derived xenograft (PDX) tumor was passaged into 12 NMRI-nu/nu mice for chemosensitivity testing, with 6 mice as untreated control group and 6 mice as treatment group. Tumor size was measured, and the relative tumor volume was determined to distinguish between sensitive and resistant NSCLC tumors. The open square with arrow indicates sample resection for targeted next-generation sequencing, DNA methylation profiling, and gene expression analyses. **b** Chemotherapeutic responsiveness of PDX to carboplatin is given as average relative tumor volume of treated to control in %. **c** Dendogram reflecting hierarchical clustering of methylation differences between PDX versus normal tissue of MeDIP- and Methyl-Seq (BS)-derived data. **d** Scatterplot of average methylation differences of six PDX normalized to its corresponding normal samples analyzed by MeDIP- and Methyl-Seq. **e** Principal component analysis of MeDIP-Seq derived data of 22 PDXs (orange), their corresponding normal lung tissues (green), and 6 primary NSCLC tumors (violet). PCAs have been computed in QSEA, based on the % methylation (beta) values of all windows overlapping promoter regions. From these, QSEA selects the 1000 most variable regions over all samples. Plotted are the first and the second components. n number

For the analyses of DNA methylation patterns of NSCLC tumors, we profiled 22 PDX models and 22 normal lung tissues obtained from the same patients, as well as six primary NSCLC tumors from which PDXs were generated (Fig. 1a, Additional file 1: Table S1).

Tumor volumes from mice treated with carboplatin were set into relation to tumors treated with solvent alone (Fig. 1a, b, Additional file 1: Table S2). Next, in order to screen for epigenetic biomarkers, which allow for identification of nonresponding patients before they received chemotherapy, we performed MeDIP-Seq analyses of all six primary NSCLC and all 22 PDX tumors and normal lung tissues (Additional file 2: Figure S2). PDXs were profiled prior to carboplatin treatment. We used the analysis package QSEA to estimate absolute methylation levels from the MeDIP-Seq enrichment data [25]. Sequence fragments originating from the mouse genome were excluded from further analysis (Additional file 2: Table S3). For a subset of samples (six PDX tumors and corresponding normal tissues), we validated the estimated methylation levels with bisulfite-based Methyl-Seq target enrichment assays covering 84 Mb of the genome including 3.7 million CpGs (Additional file 2: Table S4). Methylation levels obtained from MeDIP-Seq and Methyl-Seq were highly correlated (Spearman correlation 0.923) (Fig. 1c, d).

### PDX models amplify DNA methylation profiles of primary NSCLC tumors

**Fig. 2:**
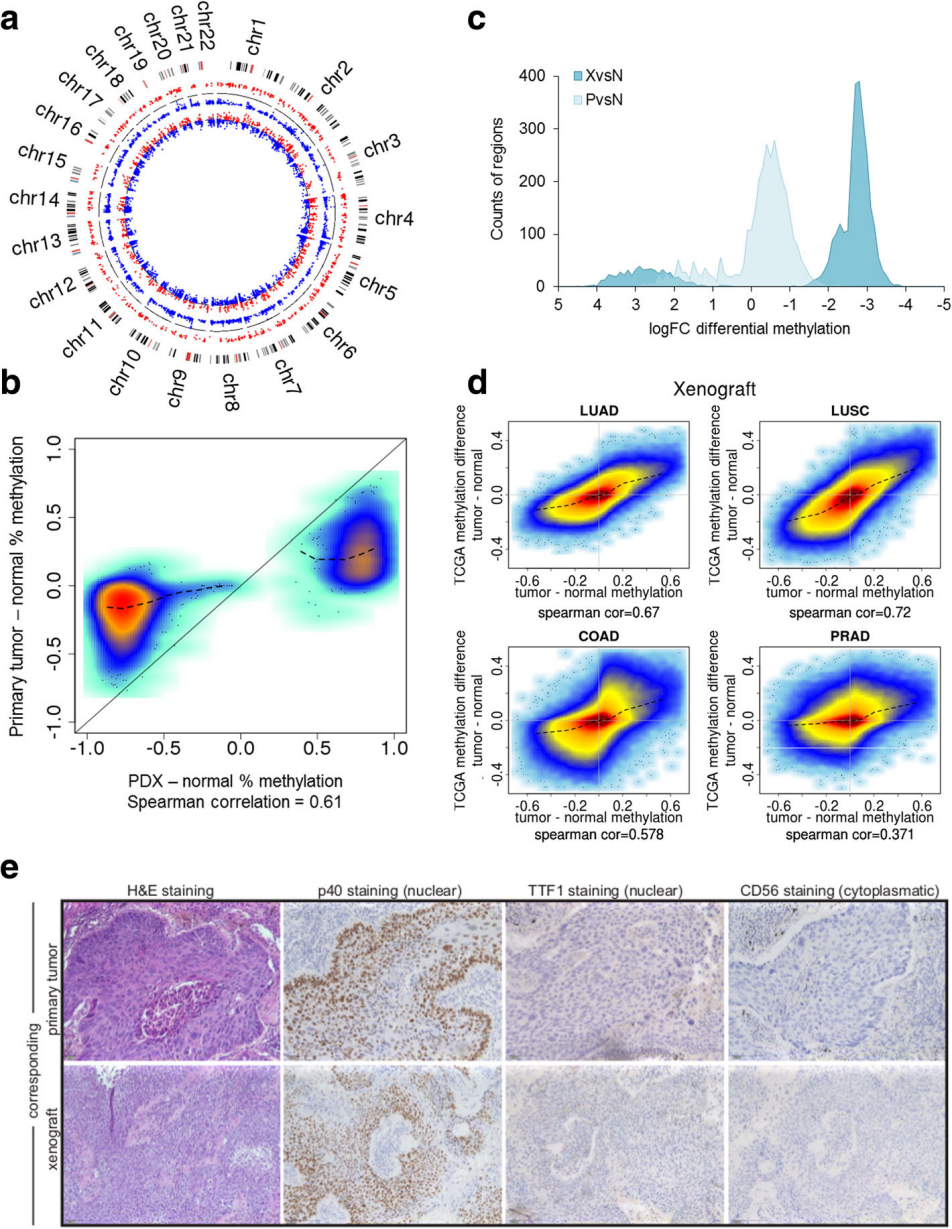
PDXs maintain DMRs of primary NSCLC tumors. **a** Circular representation of overlapping DMRs of a primary NSCLC tumor (*p* value < 0.01; inner ring) and the corresponding PDX (*p* value < 0.001; outer ring). The black line within this circle represents the baseline (zero), colored dots reflect DMRs of 250 bp window. Blue dots mark the hypomethylated and red dots the hypermethylated regions. **b** High-density scatterplot reflecting correlation of overlapping DMRs of primary NSCLC tumor methylation compared with PDX methylation. **c** Density plot with all regions detected as DMRs in PDXs. Shown are the number of differential methylations for PDXs (dark blue) and primary NSCLC (light blue) within significantly overlapping DMRs counted in PDXs (*p* value < 0.05). **d** High-density scatterplots comparing global methylation of respective histological PDXs with TCGA 450 K Arrays data LUAD, LUSC, COAD, and PRAD. LUAD lung adenocarcinoma, LUSC lung squamous carcinoma, COAD colon adenocarcinoma, PRAD prostate adenocarcinoma. **e** Immunohistochemistry of primary lung tumor and its corresponding xenograft. Representative histological (hematoxylin-eosin; H&E) and immunohistochemical (p40, TTF-1, and CD56) comparison of a squamous cell lung tumor that is positive for p40 (nuclear) and negative for TTF-1 and CD56. Scale bars 100 μm

To estimate the similarity of the methylation profiles between primary tumors and tumors generated from PDXs, we performed principal component analyses (PCA) from all MeDIP-Seq data (Fig. 1e). We observed that DNA methylation profiles separate PDX tumors and normal tissues, while primary tumors cluster between PDX and normal samples. Genome-wide Spearman correlations of the primary tumors and the corresponding PDXs ranged from 0.37 to 0.49 (Additional file 2: Figure S3 and Table S5). Thus, we wondered whether the apparent similarity of primary tumors to normal samples and the rather low genome-wide correlation values are a result of low tumor cell content in the primary tissue samples (Additional file 2: Table S5). To this end, we compared methylation alterations of PDXs and primary tumors with respect to normal tissues (Fig. 2a, b and Additional file 2: Figure S4). Most alterations in the PDXs were also observed in the corresponding primary samples albeit with a lower fold change. The mean log fold change of MeDIP enrichment within hypomethylated regions of the primary tumor is − 0.4 (FC = 0.75). For PDXs, the same regions feature more extreme changes in the same direction with a mean logFC of − 2.8 (FC = 0.14) indicating that the hypomethylation is greater in the PDXs (Fig. 2c and Additional file 2: Figure S5). Similarly, hypermethylations appear to be increased in PDXs. A well described characteristic of solid tumors is the presence of large hypomethylated blocks (LHBs), which are associated with lamina-associated domains (LADs) [37]. In order to verify the integrity of PDXs with respect to this feature, we checked for LHBs in PDXs. We detected LHBs in all samples with varying magnitude co-occurring with LADs (Additional file 2: Figure S6).

At this point, the question remained whether the PDX epigenomes truly reflect lung cancer methylomes or whether they are an outgrowth of proliferating cells without specificity for lung cancer. For this, we compared PDXs and primary tumor methylation data to Illumina 450 K array methylation data from the TCGA network for lung adenocarcinoma (LUAD, *n* = 475 tumor, 32 normal), lung squamous carcinoma (LUSC, *n* = 370 tumor, 42 normal), and other cancer cohorts (Fig. 2d) [26, 27, 38, 39]. The analysis revealed a rather high correlation of the PDXs within this study (6 adenocarcinoma and 12 squamous cell carcinoma) with the TCGA data with an average Spearman correlation of 0.72 for LUSC and 0.67 for LUAD. In contrast, other tumor types revealed a lower correlation to the PDXs, like prostate adenocarcinoma (PRAD, *n* = 512 tumor, 50 normal) with an average correlation of 0.37 and colon adenocarcinoma (COAD, *n* = 313 tumor, 38 normal) with an average correlation of 0.58. This again indicates a specific enrichment of lung cancer cells during the engraftment process, which was also apparent upon thorough histological review (Fig. 2e, Additional file 2: Table S6). Again, correlation of the primary NSCLC tumors to respective TCGA data was lower compared to PDXs supporting the low tumor content in the six primary tumors used here (squamous primary tumor (*n* = 4) with LUSC, 0.33; primary adenocarcinoma (*n* = 2) with LUAD, 0.43).

Taken together, these results indicate an enrichment of cancer cells during the engraftment process, while maintaining the tumor-specific methylation profile. In consequence, PDX samples amplify the aberrant methylation signals of primary NSCLC samples with low tumor content, facilitating the detection of individual alterations.

**Fig. 3:**
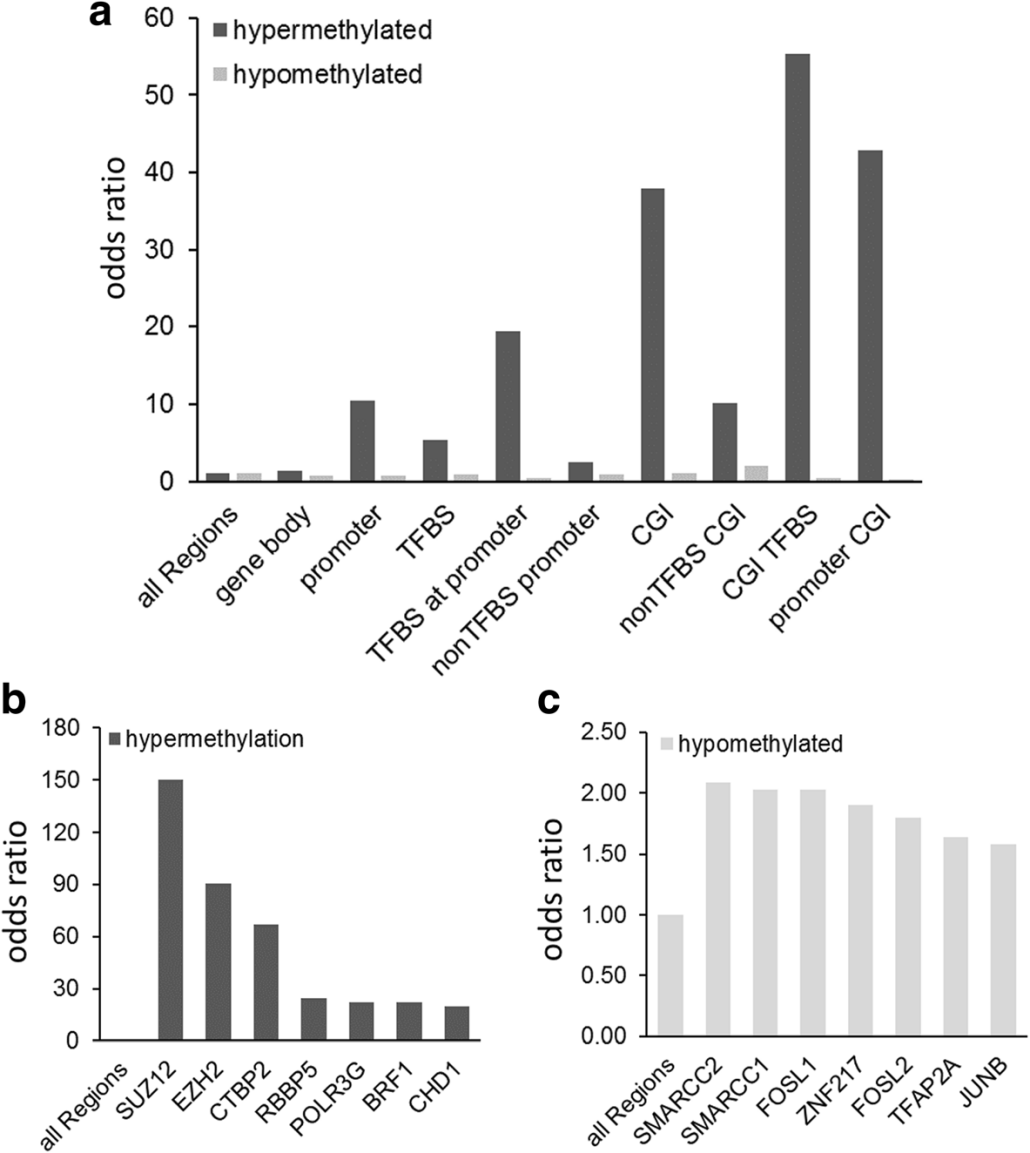
Differentially methylated regions in PDXs derived from NCSLC. **a** Barplot representing the fold enrichment of global distribution of differential hyper- and hypomethylation in PDX overlapping with regions of interest (ROIs). **b** Fold enrichment of the seven most significantly differentially methylated ENCODE-defined TFBS in PDXs for hypermethylation and in **c** for hypomethylation, respectively. The bars represent the odds ratio of the fraction of DMRs within all windows that are overlapping respective regions of interest (e.g., promoters, TFBS) over the fraction of DMRs in the whole genome. TFBS transcription factor binding site, CGI CpG island

Next, we investigated the genome-wide methylation differences between PDX and corresponding primary normal tissue. We identified 368,133 significant DMRs (adjusted *p* value < 0.0001 and methylation difference > 20%), corresponding to about 3% of all genome-wide windows. The DMRs are dominated by hypomethylation corresponding to 2.9% of the genome, whereas 0.2% is affected by hypermethylation. As expected, we found CpG islands specifically hypermethylated in PDX tumors, in particular in combination with known regulatory elements like transcription factor binding sites (TFBS) and promoters which are 55 and 43 fold enriched for hypermethylation compared to the genomic background, respectively (Fig. 3a).

As transcription factors regulate gene expression levels, and binding of transcription factors to DNA can be influenced by DNA methylation, we also analyzed ENCODE-defined TFBS for their differences in methylation levels [40]. Hypermethylated regions were highly enriched for binding sites of polycomb repressor complex 2 [41] components, such as SUZ12 and EZH2: respectively, 34 and 20.5% of all binding sites of these TFs are hypermethylated, which corresponds to an odds ratio of 149 and 90.5 (Fig. 3b). Accordingly, the promoters of several *HOX* genes (*HOXD12*, *HOXD10*), which are known to be regulated by polycomb repressor complex 2 (PRC2), were among the most significantly hypermethylated regions (data not shown), confirming previous results implying a role of DNA methylation in PRC2 dysregulation in NSCLC [42, 43]. NSCLC-specific hypomethylation in TFBS was found to be enriched for SWI/SNF chromatin remodeling factors (SMARCC1, 2) and FOS gene family members, like FOS, FOSL1, and FOSL2 (Fig. 3c). The SWI/SNF chromatin remodeling complex has already been implicated in cisplatin therapy response [44, 45]. This indicates that DNA methylation at TFBS is an important feature of cancer methylomes with a likely functional impact.

### Distinct DNA methylation alterations determine carboplatin resistance

**Fig. 4:**
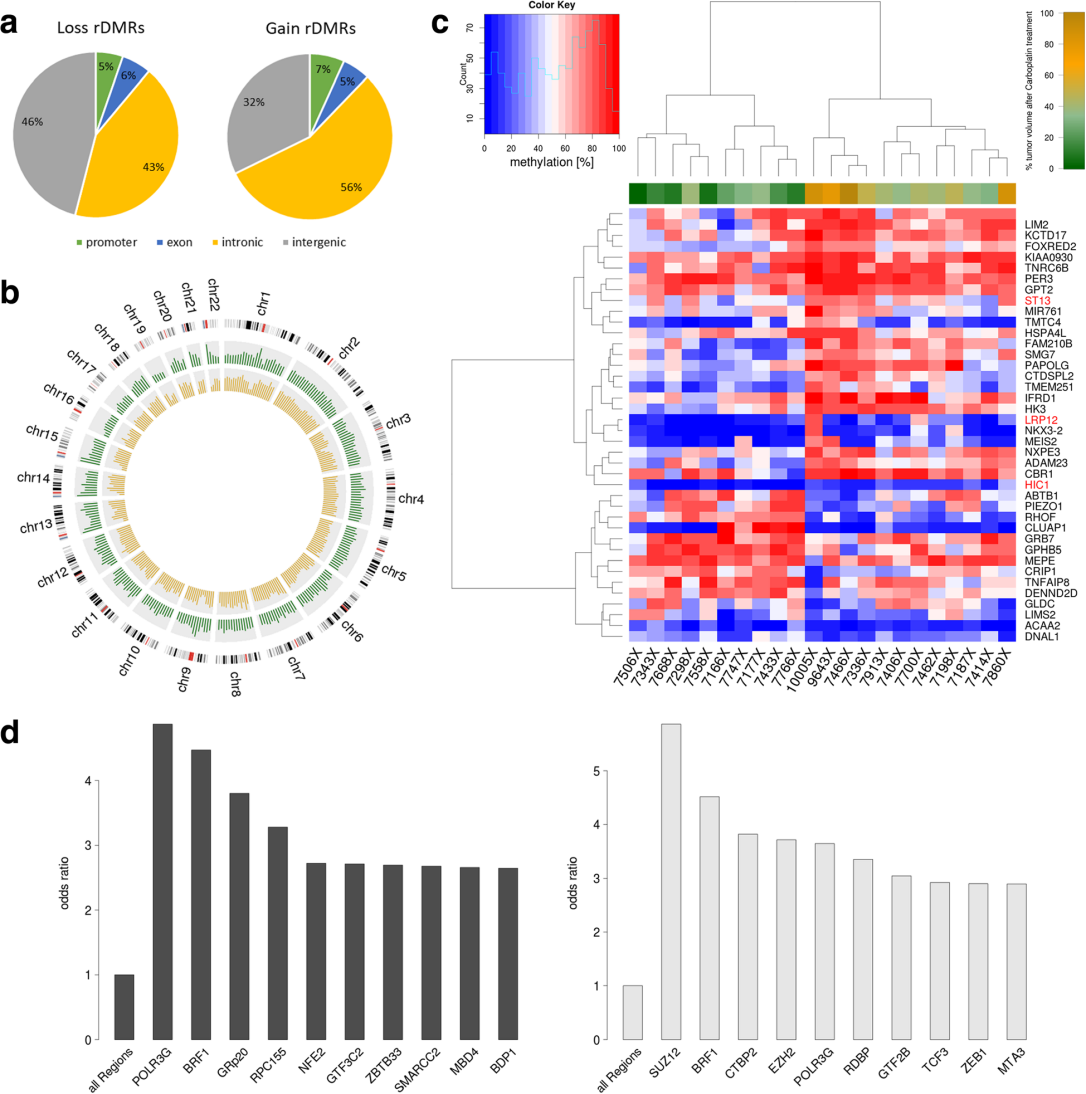
Carboplatin-resistant tumors exhibit distinct changes in DNA methylation. **a** Pie chart of the genomic distribution of carboplatin rDMRs (*p* value < 0.05). **b** Circos plot of the localization and frequency of the 837 most significantly differentially hypermethylated regions (*p* value 0.0001 and absolute correlation of methylation to relative tumor volume > 0.5). Red: hypermethylated regions in responders (263 regions); blue: hypermethylated regions in non-responders (574 regions). **c** Heatmap representation of unsupervised hierarchical clustering analysis of 40 candidate promoter-associated carboplatin rDMRs. The upper bar represents the sensitivity phenotype of the PDXs (red: non-responders; green: responders). **d** Barplot showing the most significantly differentially hyper- and hypomethylated ENCODE-defined TFBS in carboplatin non-responders. **e** Tumor vs normal methylation differences show methylation differences between non-responders and responders in large hypomethylated blocks (LHBs) on chromosome 1 as example. Green highlighted strong responder (relative tumor volume < 9%, *n* = 4), light green intermediate responder (relative tumor volume > 9%/< 30%, *n* = 5), gray weak responder (relative tumor volume > 30%/< 78%, *n* = 4), and black non-responder (relative tumor volume > 78%, *n* = 4). The dark red line indicates LADs (lamina-associated domains) that are associated in location with LHBs. TFBS transcription factor binding site, CGI CpG island

To identify resistance DMRs (rDMRs), i.e., DMRs with potential applicability as biomarkers for chemotherapy resistance, we aimed to select genomic regions where DNA methylation is associated with tumor volume after carboplatin response in PDX. Compared to the methylation difference between tumor and normal, this association is rather small and we identified only 70 rDMRs after correction for multiple testing at a relaxed FDR of 10% which were also frequently located in histocompatibility (HLA) complex regions which are difficult to target with primer-based technologies (e.g., methylation-specific PCR). In order to obtain a broader set of potential rDMRs, we used the test statistic as a score to rank potential candidates. Additionally, orthogonal locus-specific validations will then help to discriminate between true-positive and false-positive candidates. We first selected genomic windows at a *p* value of 0.01 (without correction for multiple testing) resulting in 43,065 rDMR candidates with 27,011 gains of methylation and 16,052 loss of methylation in non-responders. These candidates are mainly located within introns (50.9%) and intergenic regions (37.4%); 5.6% are associated with exons, and 6.2% with promoter regions (2678 rDMRs at promoters of 2380 genes) (Fig. 4a).

Ingenuity pathway analysis [46] of the 2380 genes revealed an enrichment of signaling pathways known to play a role in platinum drug therapy resistance, like ephrin B [47] and Wnt/ß-Catenin signaling [48], autophagy [49], EMT [50] (Additional file 2: Figure S7). Upstream regulator analysis identified genes involved in DNA methylation and transcriptional repression signaling including members of the DNA methyltransferase family (DNMT 1, 3A), histone modifiers (HDAC3), and methyl-binding proteins (MBD1, MBD2). It has recently become apparent that DNA methylation and histone modification pathways can be dependent on one another and that this crosstalk can lead to reversible and/or long-term gene repression/activation [51–53].

Furthermore, by linking rDMRs to TFBS, we found top candidates associated to platin resistance in the literature (Fig. 4d): The most significantly enriched binding sites with hypermethylation include binding sites for RNA polymerase III subunits (POLR3G, BRF1, RPC155) and binding sites for NFE2 (nuclear factor, erythroid 2) which is a paralog of NFE2L2. RNA polymerase III synthesizes small RNAs which might be involved in therapy modulation [54]. An overexpression of *NFE2L2* has been shown to be associated with cisplatin resistance in bladder and ovarian carcinoma [55, 56]. Among the most significantly enriched hypomethylated TFBS, we found the PRC2 component SUZ12 (suppressor of zeste 12 homolog). To answer whether or not the change in methylation indeed causes a change of the binding affinities of these transcription factors, additional experiments are required. However, studies exist where more than 50% of transcription factors seem to be affected by CpG methylation [46].

Next, we investigated differences between responders and non-responders in the intensity of the LHBs described above. Those LHBs with differential LHB intensity tend to be more predominant in non-responders compared to responders (e.g., on chromosome 1, 2, and 4, as shown in Additional file 2: Figure S6).

### Epigenetically driven carboplatin response genes include tumor suppressors

In order to further explore the functional impact of carboplatin rDMRs, we profiled the gene expression for 32 of the PDXs and compared promoter methylation and gene expression.

We found that for 1376 genes of the 2380 genes with promoter rDMRs, DNA methylation is correlated to tumor volume (Spearman correlation test *p* value < 0.05, Additional file 1: Table S7). Of these 1376 genes, 40 have a correlation between gene expression and tumor volume (Spearman correlation test *p* value < 0.05; Fig. 4c) which is in opposite direction to the methylation/tumor volume correlation (Additional file 1: Table S8). Interestingly, this list contains a number of genes known or suspected to encode tumor suppressors including (TSG) (http://www.uniprot.org/) *HIC1*, *LRP12*, and *ST13*.

*HIC1* (hypermethylated in cancer 1) is hypermethylated in carboplatin-resistant xenografts and is involved in the regulation of the TP53-dependent DNA damage response and of the Wnt signaling pathway. In NSCLC, a low expression is associated with shorter survival [57]. Another TP53 target, the short variant of tumor necrosis factor-α-induced protein 8 (TNFAIP8), is overexpressed in numerous human cancers, including NSCLC, and seems to repress TP53 function [58]. Here, we find the promoter significantly hypomethylated, indicating a relevance in therapy resistance of carboplatin. ST13 is involved in the heat shock response and mediates the association of HSP70 and HSP90 (heat shock proteins 70 and 90). It has been described to be downregulated in colorectal carcinoma tissue [59]. *LRP12* (LDL receptor-related protein 12) encodes a transmembrane protein that is differentially expressed in many cancer cells and that has recently been shown to be frequently hypermethylated in B cell lymphoma [60]. Downregulated *LRP12* is associated with tumorigenesis. However, its function as tumor suppressor or oncogene is controversial. In oral squamous cell carcinoma for example, Garnis et al. found *LRP12* overexpressed [61].

### LRP12 promoter methylation predicts patients’ outcome and is associated with increased overall and progression-free survival

To identify predictive biomarkers, we focused on the list of 40 genes with methylation and expression correlations to tumor volumes. Of particular interest as cancer biomarkers are tumor suppressor genes (TSGs) (http://www.uniprot.org/). In our study, the tumor suppressor gene *LRP12* (LDL receptor-related protein 12) featured among the strongest fold changes in methylation and correlation to carboplatin response with a distinct hypermethylation (log2FC 2.65, *p* value 0.009, Spearman correlation 0.52, *p* value 0.01) and downregulation in the resistant tumors. To further analyze if LRP12 is associated to carboplatin resistance, we performed siRNA knockdown experiments in NCI-H23 carboplatin-sensitive cells. Cell viability of cells with *LRP12* knockdown is significantly higher in presence of carboplatin compared to that with a control siRNA treatment (Additional file 2: Figure S8). Thus, LRP12 seems to be indeed involved in carboplatin resistance mechanisms.

To validate the methylation changes of *LRP12* and to extrapolate the finding from the PDX mouse models to clinical samples, we analyzed the methylation status of *LRP12* in an independent patient cohort by applying quantitative methylation-specific PCRs (qMSP) to formalin-fixed paraffin-embedded (FFPE) primary NSCLC tumors. This cohort consisted of 35 patients with radical (R0) surgery and adjuvant chemotherapy with cis-/carboplatin. In a period of ~ 3 years, 15 of the patients had a relapse and are thus classified as non-responders. The cohort consisted of 10 matched responder/non-responder pairs regarding tumor state, histology, age, and gender (Additional file 2: Table S9).

**Fig. 5:**
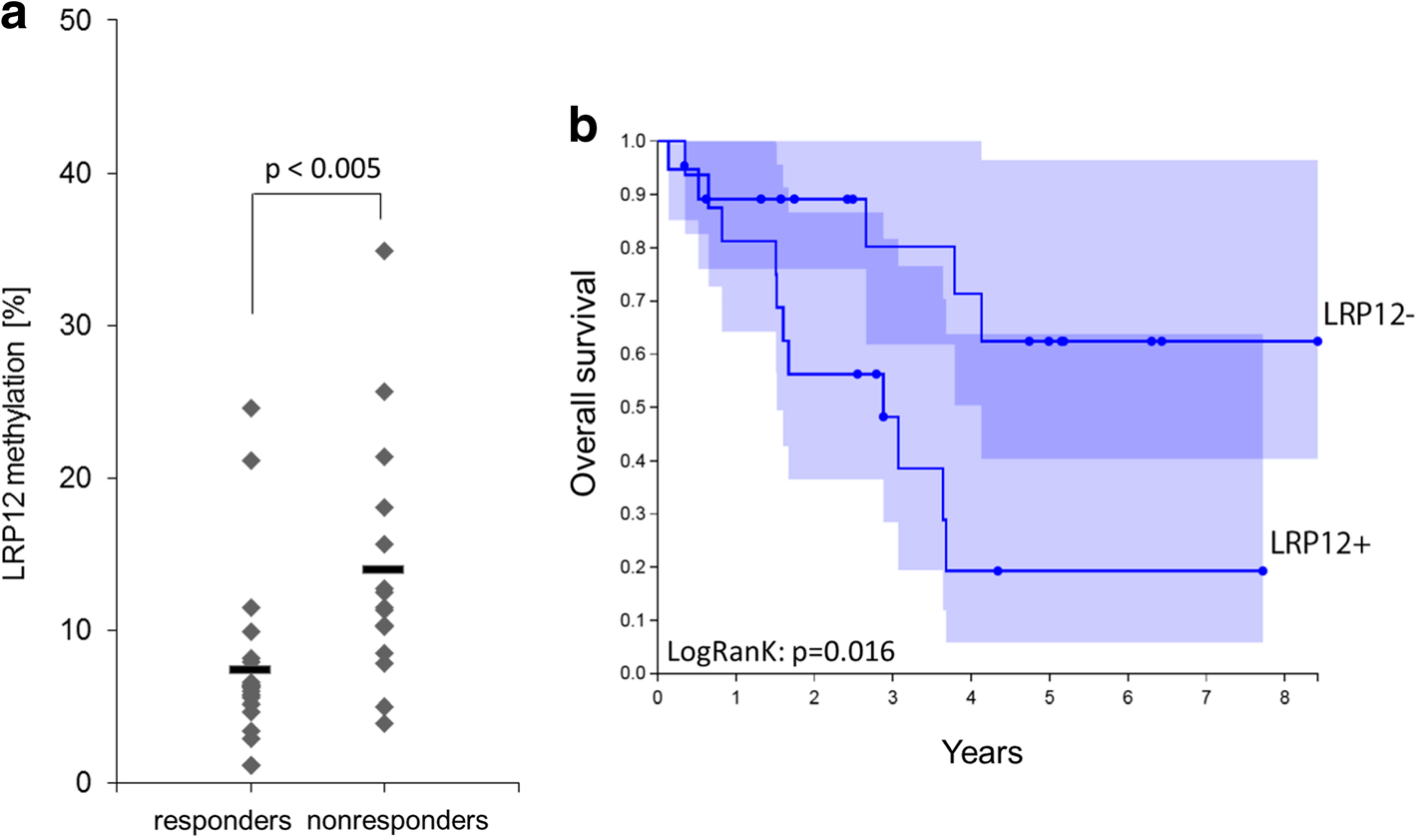
LRP12 DNA hypermethylation as independent predictive factor for clinical outcome in NSCLC. **a** *LRP12* methylation level in FFPE samples of primary NSCLC tumors of the validation cohort with relapse (non-responders, *n* = 15) or without relapse (responders, *n* = 19, conversion efficiency > 3) determined by qMSP (Mann-Whitney test, *p* < 0.003). **b** Kaplan-Meier analysis of overall survival (OS) of the same cohort as used in **a** with respect to *LRP12* methylation status (LRP12+ methylation > 8.3%, 16 patients; LRP12−, methylation < 8.3%, 19 patients). The statistical significance of the log-rank test is shown. The mean time to survival in years is indicated for each group. Confidence interval is marked in violet

*LRP12* methylation was found significantly higher in FFPE samples with relapse (non-responders) compared to that in samples without relapse (responders) (on average 13.9% vs 7.4%; Mann-Whitney test *p* value 0.003) (Fig. 5a). For the LRP12 promoter, the threshold of 8.3% methylation yielded maximum accuracy. Using this threshold, 12 out of 15 nonresponding NSCLC tumors and 16 out of 19 responding tumors are classified correctly, which corresponds to a sensitivity of 80% and a specificity of 84%.

Integrating clinical follow-up data of the same clinical validation cohort (second cohort), we generated Kaplan-Meier curves to test whether *LRP12* methylation status impacts the survival of the patients after platin therapy. As a cut-off, we used 8.3% methylation as determined from the ROC analyses (Additional file 2: Figure S9a). *LRP12* methylation status in patients classified as responders (without relapse, loss of *LRP12* methylation) correlated with a significantly higher OS (chi-square 5.8216; *p* value 0.0158) as well as a higher progression-free survival (PFS) (chi-square 5.8298; *p* value 0.0158) based on a log-rank test (Fig. 5b and Additional file 2: Figure S9b). Next, we used methylation data from 449 LUAD patients from the TCGA data set to investigate whether methylation of *LRP12* is associated to survival. Indeed, we found that high methylation (above 8.5%) results in a significant shorter survival than with low methylation of the *LRP12* promoter (Additional file 2: Figure S10). Thus, *LRP12* methylation levels provide a means for the determination of carboplatin response and progression-free survival (PFS) and OS.

## Discussion

Chemotherapeutic resistance, intrinsic or acquired, is still a major challenge in managing NSCLC therapy. In order to facilitate a personalized treatment of NSCLC patients, we aimed at identifying predictive DNA methylation biomarkers using patient-derived xenografts (PDXs). PDX models have revealed a high degree of similarity with the original clinical tumor sample in regard to histology, immunohistochemistry, as well as genomic profile. Besides this, the chemotherapeutic responsiveness of the PDX has been shown to resemble the clinical situation for different tested chemotherapeutic agents [21, 22, 62, 63], promising transferability of the obtained results.

This is a major advantage over cancer cell line models, in which previously predictive DNA methylation biomarkers were identified [12–15]. The evaluation of these biomarkers in primary NSCLC tissues revealed a weak transferability to clinical samples [15, 16].

Since the use of PDXs for cancer epigenomics is not yet comprehensively established, we investigated the similarity of the PDXs and corresponding primary tumor methylomes. We also included tumors with low tumor cell content to resemble, as much as possible, the clinical situations. Compared to previous publications that were mainly restricted to promoter methylations, we analyzed genome-wide methylations using the MeDIP-Seq technology [18, 19, 64–66]. Using the recently published QSEA method, we transformed enrichment to absolute methylation levels by considering the local CpG density as well as CNVs [25]. We validated these methylation levels using the Methyl-Seq target enrichment approach.

We observed that aberrantly methylated regions in the PDX tumors were reflected in the corresponding primary NSCLC tumors, albeit the levels of differential methylation of the PDX samples were much higher compared to the levels within the primary tumors. This effect is most likely due to a growth advantage in the PDXs, and thus, tumor cells are enriched during the engraftment process. It might also explain the increase in variant allele frequencies in PDXs compared to primary tumors [18, 67]. Similarly, Guilhamon et al. found increased methylation levels after the engraftment process at CGI shores and gene bodies [64].

We used methylation profiles of 22 PDX and corresponding normal tissue samples and identified 368,133 tumor-specific DMRs. We found hypomethylation of large blocks (LHBs) with accompanied focal hypermethylation [68–70]. Focal hypermethylations were mainly associated with promoter regions and in particular with TFBS [40]. Epigenetically affected TFBS included binding sites of PRC2 components, like SUZ12, EZH2, RNA polymerase III, and FOS family members. Interestingly, we found FOS target sites to be mainly hypomethylated which would prevent FOS binding and which might function as regulatory mechanism counter-acting cellular proliferation [71].

We used chemotherapeutic response data obtained by chemosensitivity testing in the PDXs to characterize tumor samples according to their carboplatin sensitivity phenotype. We identified differentially methylated regions associated with therapy response (rDMRs). These regions are enriched for hypermethylated *SMARC* binding sites in the nonresponding PDX. *SMARC* proteins belong to the SWI/SNF chromatin remodeling complex and have been associated with resistance to platinum-based drugs like cisplatin [72–74].

Enrichment analysis of the subset of the 2380 promoter-associated rDMRs revealed signaling pathways known to be involved in platinum resistance, like ephrin B and Wnt/β-catenin signaling. Upstream analyses and enrichment analyses of transcription factor binding sites showed DNA methylation, histone methylation, and chromatin re-modeling to be involved in carboplatin therapy resistance on several levels.

Finally, we identified genomic regions where DNA methylation levels are correlated to carboplatin response and suggest these regions as candidates for predictive biomarkers. Validation of the candidates in an independent cohort of primary NSCLC patients confirms the potential of *LRP12* as predictive biomarker of platinum-based therapy in NSCLC.

Thus, the outlined strategy from patient’s tumor material over PDX models, epigenome profiling, and validation in an independent cohort renders a straightforward approach to extract clinically relevant epigenetic markers. The detour over PDXs enables an accurate acquisition of chemotherapy response rates in a genetic homogenous background even with monotherapies, which is impossible to achieve by a restriction to patient’s tissue materials only.

## Conclusions

We have identified a candidate epigenetic predictive biomarker for platin therapy resistance in NSCLC. The sensitivity and specificity we achieved exceeds other predictive biomarkers and was validated on a second, independent cohort of patients. However, additional validations are required for an estimation of the usability. Tissue material used was formalin-fixed and paraffin embedded (FFPE), thus immediately mirroring the clinical situation and improving the applicability of the markers. Our experiments were initiated with epigenome-wide profiles of NSCLC PDXs which were treated with chemotherapy or solvent alone as control. Since the genetic background of the PDXs is identical and the response rate can be quantified, it makes PDXs ideal for chemical compound tests. Additionally, we show that the tumor cells are outgrown in the PDXs which significantly facilitate the detection of epigenetic alterations. Based on these findings, other chemotherapies are queried for potent epigenetic predictive markers. Next urgent steps include an integration of the *LRP12* epigenetic test into the Network Genomic Medicine (NGM) Lung Cancer platform, Europe’s largest platform for molecular testing, to improve the accuracy of the prediction of platin therapy resistance and a transfer into clinical applications.

## Additional files

Additional file 1: **Table S1.** Clinical characteristics of the study cohort. **Table S2.** Response rates of PDXs to carboplatin treatment. **Table S7.** Carboplatin therapy resistance-associated genes for which promoter DNA methylation is correlated to tumor volume. **Table S8.** Tumor suppressor genes with significant carboplatin rDMRs and differential expression. (DOC 4660 kb)

Additional file 2: Additional Methods. **Figure S1.** Region used for the MSP of *LRP12.* **Figure S2.** MeDIP-Seq statistics of 54 samples including primary tumor tissues (P) and PDXs (X). **Table S3.** MeDIP-Sequencing statistics. Table S4 Methyl-Sequencing statistics. **Figure S3.** Correlation of methylation of overlapping DMRs in primary NSCLC and patient-derived xenografts. **Table S5.** Tumor content of primary tumors and genome-wide Spearman correlation of DMRs of primary tissue to the PDXs. **Figure S4.** Patientwise circos plots of overlapping DMRs of primary NSCLC and PDX. **Figure S5.** Comparison of methylation values of primary NSCLCs and PDXs. **Figure S6.** Methylation differences between non-responders and responders in large hypomethylated blocks (LHBs) on chromosomes 1, 2, and 4 as examples. **Table S6.** Histopathologic evaluation of primary tumor and PDX tumor. **Figure S7.** Ingenuity pathway and upstream regulator analyses of the 2380 genes differentially methylated. **Figure S8.** *LRP12* knockdown induces carboplatin resistance. **Table S9.** Patient’s data and clinical characteristics of the validation cohort. **Figure S9.** *LRP12* DNA hypermethylation as independent factor predictive for clinical outcome in NSCLC. Figure S10 *LRP12* DNA hypermethylation as independent predictive factor for clinical outcome in 449 NSCLC patients from the TCGA data set. Additional references. (XLSX 141 kb)

## Abbreviations

COAD: Colon adenocarcinoma
CNV: Copy number variations
DAPK: Death-associated protein kinase
DMR: Differentially methylated region
EGFR: Epidermal growth factor receptor
FDR: False discovery rates
FFPE: Formalin-fixed and paraffin-embedded
GLM: Generalized linear model
IGFBP3: Insulin-like growth factor-binding protein 3
IPA: Ingenuity Pathway Analysis
LAD: Lamina-associated domain
LCN2: Lipocalin 2
LHB: Large hypomethylated block
LRP12: LDL receptor-related protein 12
LUAD: Lung adenocarcinoma
LUSC: Lung squamous carcinoma
MeDIP: Methylated DNA immunoprecipitation
NSCLC: Non-small cell lung cancer
OS: Overall survival
PDX: Patient-derived xenograft
PFS: Progression-free survival
PRAD: Prostate adenocarcinoma
PRC2: Polycomb repressor complex 2
qMSP: Quantitative methylation-specific PCR
rDMR: DMR-associated with therapy response
ROC: Receiver operating characteristic analysis
SLFN11: Schlafen family member 11
SUZ12: Suppressor of zeste 12 homolog
TCGA: The Cancer Genome Atlas
TFBS: Transcription factor binding sites
TGM2: Transglutaminase 2
TSGs: Tumor suppressor genes
