# Supplementary material for: Epigenomic profiling of non-small cell lung cancer xenografts uncover LRP12 DNA methylation as predictive biomarker for carboplatin resistance
Source: Genome Med. 2018 Jul 20;10:55. doi: 10.1186/s13073-018-0562-1 (PMC6054719; doi:10.1186/s13073-018-0562-1)
Supplement: Supplementary file 2 — Additional Methods. Figure S1. Region used for the MSP of LRP12. Figure S2. MeDIP-Seq statistics of 54 samples including primary tumor tissues (P) and PDXs (X). Table S3. MeDIP-Sequencing statistics. Table S4 Methyl-Sequencing statistics. Figure S3. Correlation of methylation of overlapping DMRs in primary NSCLC and patient-derived xenografts. Table S5. Tumor content of primary tumors and genome-wide Spearman correlation of DMRs of primary tissue to the PDXs. Figure S4. Patientwise circos plots of overlapping DMRs of primary NSCLC and PDX. Figure S5. Comparison of methylation values of primary NSCLCs and PDXs. Figure S6. Methylation differences between non-responders and responders in large hypomethylated blocks (LHBs) on chromosomes 1, 2, and 4 as examples. Table S6. Histopathologic evaluation of primary tumor and PDX tumor. Figure S7. Ingenuity pathway and upstream regulator analyses of the 2380 genes differentially methylated. Figure S8. LRP12 knockdown induces carboplatin resistance. Table S9. Patient’s data and clinical characteristics of the validation cohort. Figure S9. LRP12 DNA hypermethylation as independent factor predictive for clinical outcome in NSCLC. Figure S10 LRP12 DNA hypermethylation as independent predictive factor for clinical outcome in 449 NSCLC patients from the TCGA data set. Additional references. (XLSX 141 kb) [file 13073_2018_562_MOESM2_ESM.doc]

**Additional Methods**

**Cell culture and siRNA knock down of *LRP12***

The carboplatin sensitive non-small-cell lung cancer cell line NCI-H23 (CRL-5800, ATCC; Bar et al., 2016) was cultivated at 37 °C and 5% CO2 in RPMI (#21875034, Life Technologies) supplemented with 10% fetal bovine serum (FBS, #F7524, SIGMA-Aldrich) and 100 U/ml penicillin/streptomycin (Biochrom). Cells were regularly tested for mycoplasma contamination using the MycoAlert PLUS detection kit (Lonza) and were proven to be mycoplasma free. The *LRP12* siRNA pool (ON-Targetplus human SmartPool, #L-010233-00), the non-targeting siRNA control pool (#L-005033-00) as well as the miRIDIAN microRNA Mimic Transfection Control with Dy547 (#CP-004500-01) were purchased from Dharmacon.

NCI-H23 cells (8x10^5^) were seeded in a well of a 24 well plate. After 24h cells were transfected with Lipofectamine 3000 (Life Technologies) and treated with 25µg/ml Carboplatin (#13112, Bertin Pharma). Transfection efficiency was monitored using the miRIDIAN microRNA Mimic Transfection Control with Dy547 in a separate well and was usually around 80 %.

Knock down efficiencies were determined with quantitative PCR using primers targeting *LRP12* and *HPRT*:

LRP12F 5’-tacctccagcttctcctccc-3’

LRP12R 5’-agcaccatttccgtacaccc-3’

HPRT_F 5’-aggaaagcaaagtctgcattg-3’

HPRT_R 5’-ggtggagatgatctctcaact-3’

Expression was calculated by comparative quantification with the delta delta Ct method. *LRP12* expression was normalized to the house keeping gene *HPRT*.

**Neutral red assay**

Cell viability was measured 72h after transfection using the neutral red assay as described (Repetto et al., 2008). In brief, cells were incubated for 2,5 h with medium containing neutral red (#N4638, Sigma). After removal of medium the paltes were washed once with PBS. The dye was extracted with acidified neutral red destain solution by shaking the plate rapidly at 200 rpm for at least 15 minutes and 37°C. After forming a homogeneous solution, 100 µl per well were pipetted in duplicates in a 96 well corning plate. The fluorescence was read using a spectrophotometer with wavelengths of 530 and 645 nm.

**Figure S1: Region used for the MSP of *LRP12*.**

Shown is the region surrounding the MSP product for *LRP12*. Blue: localization of the MSP primer pairs, red: CpGs interrogated by MSP, green: cytosines which are prone to bisulfite conversion and are thus considered in the MSP primer sequence.

hg19_dna range=chr8:105600151-105600600

5’AAGAACGGTGAAGTCAGAAGTGTAACTTAATTATCTTAAGAGATAGATGTTCTTTGCAATTAATTCCATTCAGCTCTTGCTCAGACCCCAACGCAATATAAAGCTGTC**CG**CACAGTCTTCAGCCTTCTCTTAACC**CG**AAAGGAGTTATCTCTAACC**CG**ATCCAT**CG**ACTGGAATAAAGTAACAGTGACCACAG**CG**G**CG**GA**CG**AGGATGGATGAAATTACAATTGAACTAGGAGTCAGGACAGATGTGTACACCCTAACAGGAGAGACTGAGGTGGGGCTGGAAACTATTTTCTGGCCTTCTCC**CGCG**TGCTGCTCC**CG**GGGAACCAACAGGACCCTGCA**CG**CC**CG**GATTCGGCAGAGCCCTTGCCCCCGAGGTGGAGTGGGGAGAAGTCAGCGGGAAGCCTTTTCAGGGAATCGAGTAGGACAAGGGCTGGCGCGGTGGGAATCTCGCCG3’

**Figure S2: MeDIP-Seq statistics of 54 samples including primary tumor tissues (P) and PDXs (X).**

The processed sample reads were aligned to both graft (GRCm38/mm10) and host (GRCh37/hg19). X, PDX; N, normal; P, primary NSCLC tissue sample; n.d., not determined.

**
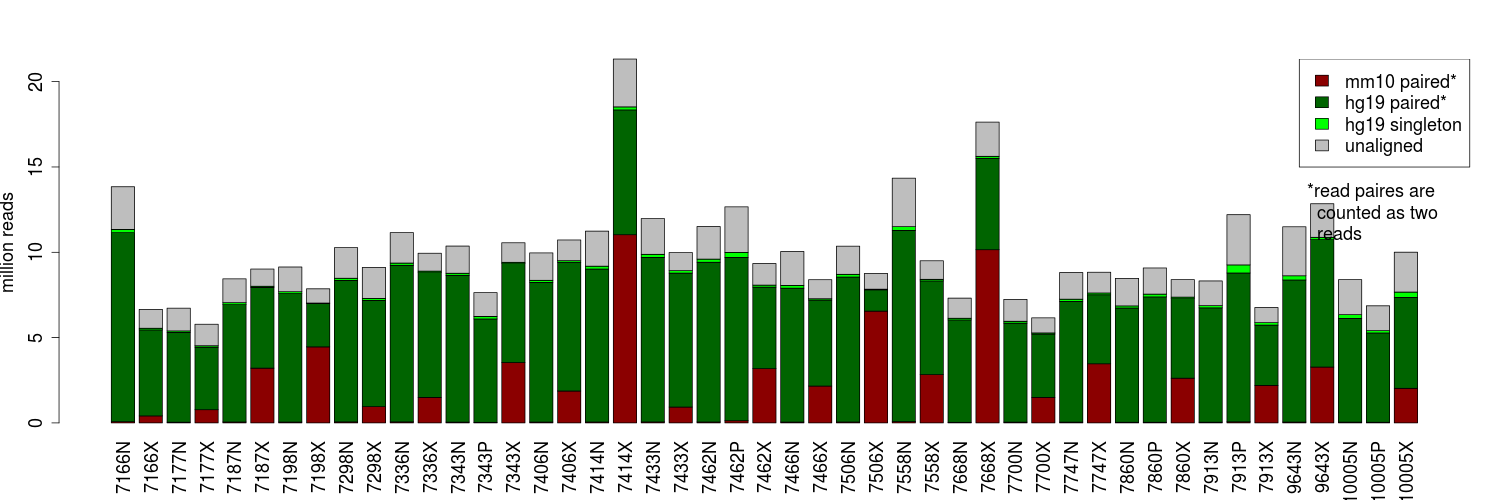
**

**Table S3: MeDIP-Sequencing statistics.**

The processed sample reads were aligned to both graft (human GRCh37/hg19 reference genome, UCSC version, Feb 2009) and host (GRCm38/mm10 mouse genome (UCSC version, July 2007) genomes.

| **samples** | **run** | **total_reads** | **mm10** | **hg19_pair** | **hg19_single** | **unaligned** |
| --- | --- | --- | --- | --- | --- | --- |
| 7166N | 3N_MRS446_ATCACG_L002 | 138411264 | 768940 | 110793402 | 1841839 | 25007083 |
| 7166X | 3T_MRS447_ACTTGA_L002 | 66536176 | 4102293 | 50430728 | 947224 | 11055931 |
| 7177N | 4N_MRS448_TAGCTT_L002 | 67253654 | 446858 | 52551840 | 936407 | 13318549 |
| 7177X | 4T_MRS449_GGCTAC_L002 | 57830206 | 7741503 | 36646030 | 846463 | 12596210 |
| 7187N | 5N_MRS403_ATCACG_L001 | 84439922 | 568708 | 68801816 | 1102102 | 13967296 |
| 7187X | 5T_MRS403_ACTTGA_L001 | 90177122 | 32111990 | 47266864 | 718222 | 10080046 |
| 7198N | 6N_MRS403_TAGCTT_L001 | 91370022 | 517358 | 75307322 | 1071492 | 14473850 |
| 7198X | 6T_MRS403_GGCTAC_L001 | 78598728 | 44569805 | 25321151 | 371539 | 8336233 |
| 7298N | 7N_MRS474_ATCACG_L001 | 102708018 | 621398 | 82849285 | 1323752 | 17913583 |
| 7298X | 7T_MRS475_ACTTGA_L001 | 91131556 | 9558274 | 62214326 | 1227104 | 18131852 |
| 7336N | 8N_MRS476_TAGCTT_L001 | 111506336 | 619902 | 91745767 | 1313583 | 17827084 |
| 7336X | 8T_GGCTAC_L001 | 99432960 | 14916321 | 73186387 | 848423 | 10481829 |
| 7343N | 9N_MRS438_ATCACG_L004 | 103633640 | 492694 | 86012908 | 1199024 | 15929014 |
| 7343N_2 | mpg_L6672-1_MRS804_S25 | 72722864 | 383386 | 56715908 | 1644990 | 13978580 |
| 7343P | mpg_L6670-1_MRS802_S23 | 76347748 | 360250 | 60553644 | 1468767 | 13965087 |
| 7343X | 9T_MRS439_ACTTGA_L004 | 105561216 | 35349749 | 58004270 | 758108 | 11449089 |
| 7343X_2 | mpg_L6671-1_MRS803_S24 | 81190746 | 29758823 | 40067688 | 980040 | 10384195 |
| 7406N | 11N_MRS440_TAGCTT_L004 | 99648938 | 512168 | 81781116 | 1292171 | 16063483 |
| 7406X | 11T_MRS441_GGCTAC_L004 | 107207434 | 18701804 | 75497949 | 967091 | 12040590 |
| 7414N | 12N_MRS442_ATCACG_L005 | 112395360 | 564992 | 89623890 | 1676898 | 20529580 |
| 7414X | 12T_merged | 213239714 | 110289893 | 73151925 | 1774690 | 28023206 |
| 7414X_1 | 12T_MRS443_ACTTGA_L005 | 88789448 | 45846659 | 30461745 | 727266 | 11753778 |
|  |  |  |  |  |  |  |
| **samples** | **run** | **total_reads** | **mm10** | **hg19_pair** | **hg19_single** | **unaligned** |
| 7414X_2 | 12T_MRS443_ACTTGA_L008 | 124450266 | 64443234 | 42690180 | 1047424 | 16269428 |
| 7433N | 13N_MRS444_TAGCTT_L005 | 119755972 | 591822 | 96389985 | 1822295 | 20951870 |
| 7433X | 13T_MRS445_GGCTAC_L005 | 99861732 | 9305562 | 78484747 | 1478037 | 10593386 |
| 7462N | 14N_MRS450_ATCACG_L003 | 115083812 | 565112 | 93614494 | 1833896 | 19070310 |
| 7462P | mpg_L6681-1_MRS813_S34 | 126629982 | 1294512 | 95735778 | 2873464 | 26726228 |
| 7462X | 14T_MRS451_ACTTGA_L003 | 93401676 | 31851209 | 47754252 | 1152096 | 12644119 |
| 7466N | 15N_MRS452_TAGCTT_L003 | 100464874 | 528614 | 78439379 | 1635607 | 19861274 |
| 7466X | 15T_MRS453_GGCTAC_L003 | 83932702 | 21612630 | 50394076 | 773457 | 11152539 |
| 7506N | 16N_MRS_462_ATCACG_L005 | 103584770 | 562658 | 84929716 | 1609060 | 16483336 |
| 7506X | 16T_MRS_463_ACTTGA_L005 | 87576354 | 65528783 | 12481820 | 376006 | 9189745 |
| 7530N | 17N_MRS_464_TAGCTT_L005 | 89422878 | 482672 | 75697447 | 3258449 | 9984310 |
| 7530X | 17T_MRS_465_GGCTAC_L005 | 90446028 | 28908628 | 49176494 | 1785397 | 10575509 |
| 7558N | 18N_MRS_466_ATCACG_L006 | 143419564 | 774506 | 112037284 | 2245200 | 28362574 |
| 7558X | 18T_MRS_467_ACTTGA_L006 | 95023096 | 28305844 | 54921280 | 918515 | 10877457 |
| 7612N | 19N_MRS_468_TAGCTT_L006 | 91793068 | 567772 | 74082303 | 1457906 | 15685087 |
| 7612X | 19T_MRS_469_GGCTAC_L006 | 79439032 | 9904504 | 58316265 | 936336 | 10281927 |
| 7668N | 20N_MRS_454_ATCACG_L004 | 73124526 | 360144 | 60005625 | 976637 | 11782120 |
| 7668X | 20T_merged | 176291158 | 101507620 | 53562899 | 1178771 | 20041868 |
| 7668X_1 | 20T_MRS_455_ACTTGA_L004 | 52833126 | 30319277 | 16262397 | 330515 | 5920937 |
| 7668X_2 | 20T_MRS_455_ACTTGA_L005 | 123458032 | 71188343 | 37300502 | 848256 | 14120931 |
| 7700N | 21N_MRS_456_TAGCTT_L004 | 72404492 | 477236 | 58004662 | 1086112 | 12836482 |
| 7700X | 21T_MRS_457_GGCTAC_L004 | 61580272 | 14964543 | 37147734 | 631619 | 8836376 |
| 7747N | 22N_merged | 88132466 | 473472 | 70724005 | 1329001 | 15605988 |
| 7747N_1 | 22N_MRS_458_ATCACG_L003 | 36549974 | 197236 | 29515294 | 468347 | 6369097 |
| 7747N_2 | 22N_MRS_458reseq_ATCACG_L008 | 51582492 | 276236 | 41208711 | 860654 | 9236891 |
| 7747X | 22T_merged | 88312728 | 34669688 | 40449817 | 1043071 | 12150152 |
| 7747X_1 | 22T_MRS_459_ACTTGA_L003 | 32562774 | 12822565 | 15030455 | 339699 | 4370055 |
|  |  |  |  |  |  |  |
| **samples** | **run** | **total_reads** | **mm10** | **hg19_pair** | **hg19_single** | **unaligned** |
| 7747X_2 | 22T_MRS_459reseq_ACTTGA_L008 | 55749954 | 21847123 | 25419362 | 703372 | 7780097 |
| 7766N | 23N_merged | 92133252 | 587222 | 70660644 | 1818852 | 19066534 |
| 7766N_1 | 23N_MRS_460_TAGCTT_L003 | 37163216 | 236608 | 28674894 | 657276 | 7594438 |
| 7766N_2 | 23N_MRS_460reseq_TAGCTT_L008 | 54970036 | 350614 | 41985750 | 1161576 | 11472096 |
| 7766P | MRS_531_ATCACG_L006 | 110030468 | 777756 | 86675979 | 2341294 | 20235439 |
| 7766X | 23T_merged | 99405744 | 18495725 | 62959414 | 2001232 | 15949373 |
| 7766X_1 | 23T_MRS_461_GGCTAC_L003 | 35432168 | 6565523 | 22542099 | 681349 | 5643197 |
| 7766X_2 | 23T_MRS_461reseq_GGCTAC_L008 | 63973576 | 11930202 | 40417315 | 1319883 | 10306176 |
| 7860N | 24N_MRS_470_ATCACG_L007 | 84704552 | 394814 | 66947396 | 1233499 | 16128843 |
| 7860P | MRS_532_ACTTGA_L006 | 90812034 | 381244 | 73467490 | 1634840 | 15328460 |
| 7860X | 24T_MRS_471_ACTTGA_L007 | 83982882 | 26226962 | 46639127 | 862695 | 10254098 |
| 7913N | 25N_MRS_472_TAGCTT_L007 | 83203680 | 451690 | 66976327 | 1334644 | 14441019 |
| 7913P | MRS_534_GGCTAC_L006 | 122040074 | 656606 | 87198414 | 4738283 | 29446771 |
| 7913X | 25T_MRS_473_GGCTAC_L007 | 67619300 | 22018148 | 35308994 | 1471378 | 8820780 |
| 9643N | MRS494_TAGCTT_L004 | 114935874 | 521270 | 83244104 | 2476110 | 28694390 |
| 9643X | MRS495_GGCTAC_L004 | 128444756 | 32739421 | 74709926 | 1322217 | 19673192 |
| 10005N | mpg_L6676-1_MRS808_S29 | 83955946 | 489720 | 60763770 | 2252264 | 20450192 |
| 10005P | mpg_L6674-1_MRS806_S27 | 68636776 | 401050 | 52313922 | 1380976 | 14540828 |
| 10005X | mpg_L6675-1_MRS807_S28 | 100041620 | 20268736 | 53241158 | 3177492 | 23354234 |
| 10684N | mpg_L6678-1_MRS810_S31 | 31436648 | 174736 | 22819416 | 676813 | 7765683 |
| 10684P | mpg_L6680-1_MRS812_S33 | 94511988 | 36296862 | 37327917 | 1278322 | 19608887 |
| 10684X | mpg_L6679-1_MRS811_S32 | 107831154 | 2952457 | 70721533 | 5367554 | 28789610 |
| A549 | MRS539_ATCACG_L007 | 89553814 | 475570 | 71425440 | 2069631 | 15583173 |
| H1299 | MRS_537_TAGCTT_L007 | 110873212 | 467416 | 89372179 | 2740406 | 18293211 |
| H1650 | MRS_538_GGCTAC_L007 | 163422496 | 757048 | 137327761 | 2423323 | 22914364 |
| HCC827 | MRS_535_ATCACG_L007 | 90930820 | 393378 | 69414247 | 2450781 | 18672414 |

mm10, mouse reference genome GRCm38/mm10; hg19, human reference genome RCh37/hg19, N, normal tissue, X, patient-derived xenograft, P, primary tumor**.**

**Table S4: Methyl-Sequencing statistics.**

The processed sample reads were aligned to both graft (human GRCh37/hg19 reference genome, UCSC version, Feb 2009) and host (GRCm38/mm10 mouse genome (UCSC version, July 2007) genomes.

| **Sample** | **Total reads** | **mm10** | **hg19 paired reads** | **hg19 ambiguous reads** | **unaligned reads** |
| --- | --- | --- | --- | --- | --- |
| 7336N | 50092405 | 119732 | 45023569 | 1589157 | 3479679 |
| 7336X | 54856434 | 942013 | 49274490 | 2250275 | 3331669 |
| 7177N | 42563077 | 89947 | 38717067 | 958640 | 2887370 |
| 7177T | 43389538 | 480962 | 38723006 | 1562361 | 3104171 |
| 7187N | 44046687 | 97611 | 40211313 | 939180 | 2896194 |
| 7187T | 40164922 | 1099605 | 35334953 | 1835712 | 2994257 |
| 7860P | 21493161 | 47720 | 19602381 | 515509 | 1375271 |
| 7913N | 21785528 | 48050 | 19873371 | 546306 | 1365851 |
| 7913X | 22119079 | 564253 | 19400553 | 1050146 | 1668380 |
| 7913P | 27937945 | 89314 | 25538826 | 638152 | 1760967 |
| 7198N | 26669696 | 60178 | 24316267 | 622896 | 1730533 |
| 7198X | 20267529 | 1768383 | 16295960 | 2128905 | 1842664 |
| 7860N | 23481238 | 55874 | 21426249 | 629272 | 1425717 |
| 7860X | 32926686 | 841784 | 28801882 | 1604784 | 2520020 |

N, normal tissue, X, patient-derived xenograft, P, primary tumor**.**

**Figure S3: Correlation of methylation of overlapping DMRs in primary NSCLC and patient-derived xenografts.**

Patientwise high density scatterplots reflecting the correlation of overlapping differentially methylated regions (DMRs) of primary NSCLC tumor methylation compared to patient-derived xenograft (PDX) methylation. Spearman correlation coefficients are indicated below each figure.


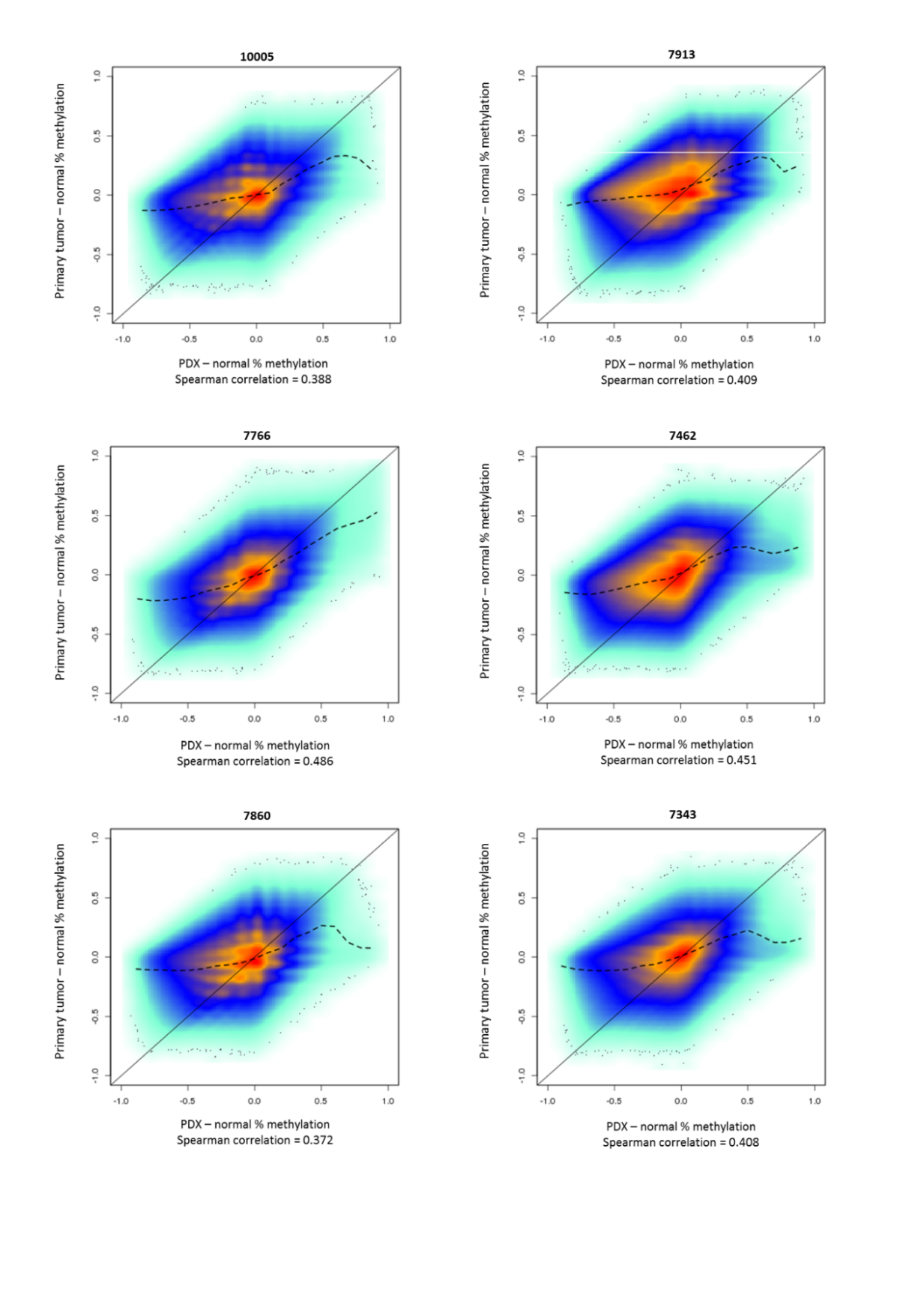


**Table S5: Tumor content of primary tumors and genome-wide Spearman correlation of DMRs of primary tissue** to the PDXs.

| **Sample** | **Tumor content [%]** | **Spearman correlation** |
| --- | --- | --- |
| 7343 | 70 | 0.408 |
| 7766 | 70 | 0.486 |
| 7860 | 30 | 0.372 |
| 7913 | 5 | 0.409 |
| 10005 | 10 | 0.388 |
| 7462 | n.d. | 0.451 |

**Figure S4: Patientwise circos plots of overlapping DMRs of primary NSCLC and PDX.**

Circular representation of the overlapping DMRs of the primary NSCLC tumor vs normal (p-value < 0.01; second innermost ring) and the corresponding PDX vs normal (p-value < 0.001; innermost ring. Black line represents the baseline (zero) and colored dots represent 250bp window DMRs. Blue dots mark hypomethylated and red dots hypermethylated regions.


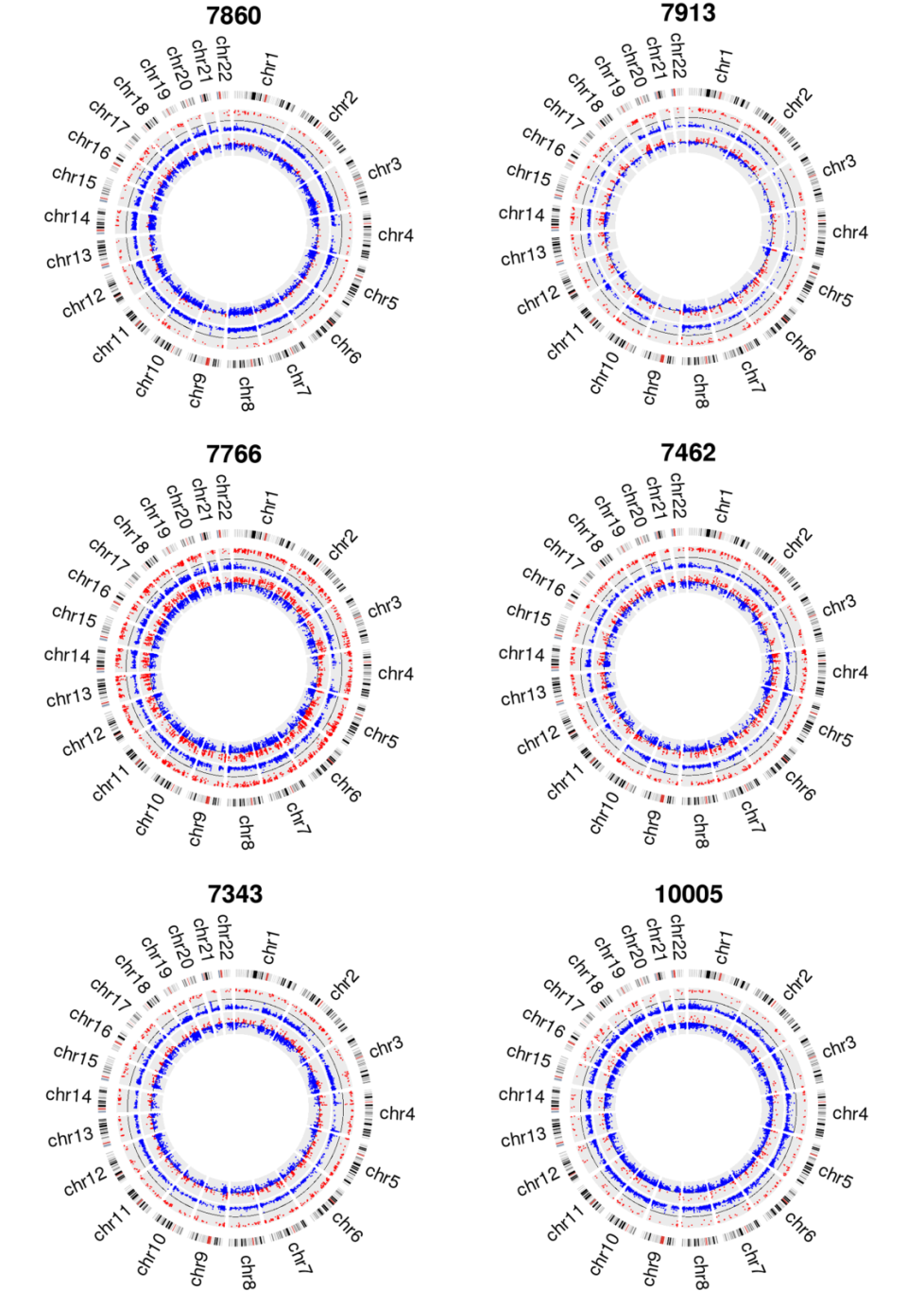


**Figure S5. Comparison of methylation values of primary NSCLCs and PDXs.**

Patientwise comparison of the range of methylation levels in DMRs (p-value < 0.05) calculated from MeDIP-Seq data in primary NSCLC and the corresponding PDX. XvsN, PDX versus normal tissue; PvsN, primary tumor versus normal tissue.

**
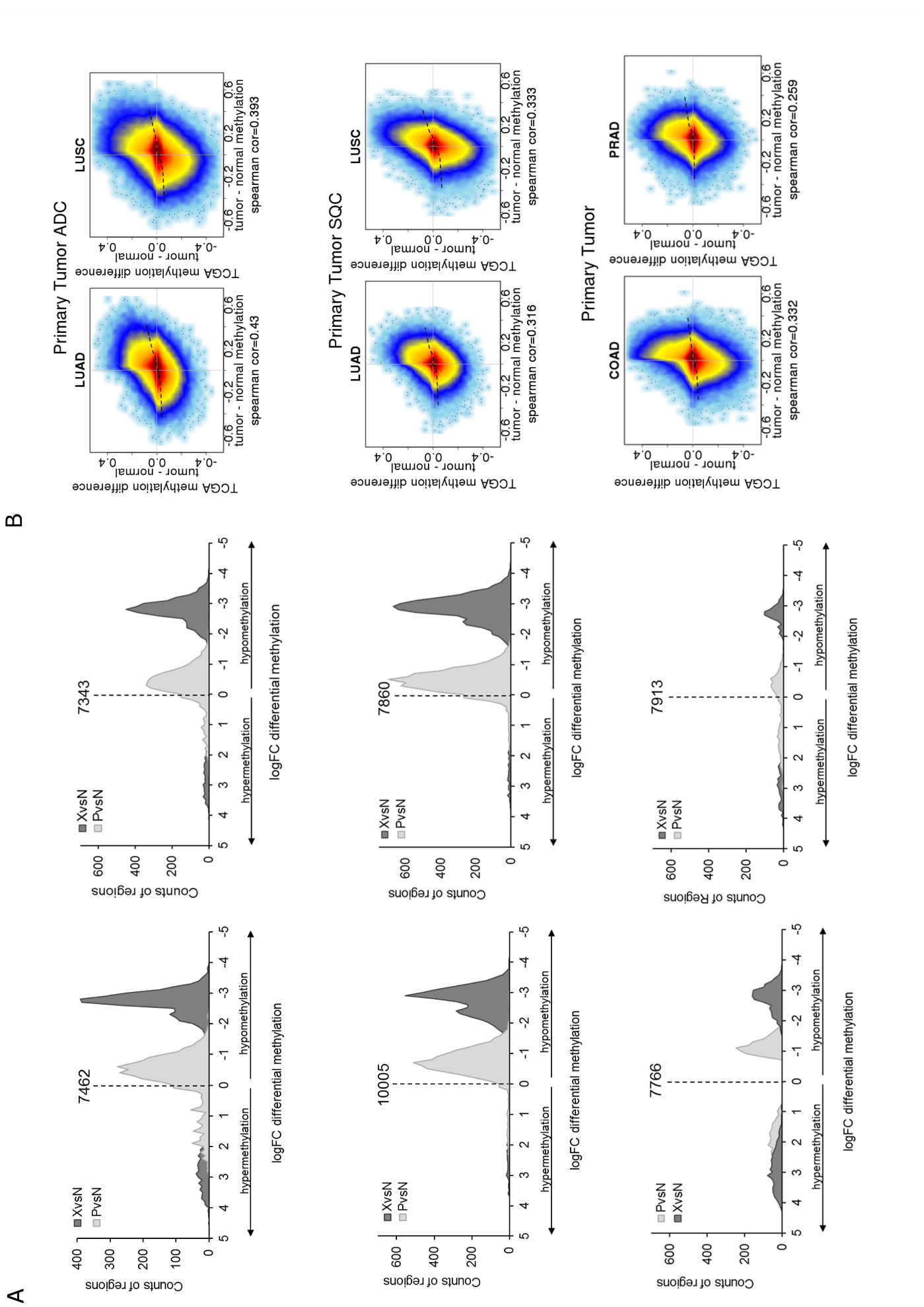
**

**Figure S6: Methylation differences between non-responders and responders in large hypomethylated blocks (LHB) on chromosome 1, 2 and 4 as examples**.

Green highlighted strong responder (relative tumor volume < 9%, n = 4), light green intermediate responder (relative tumor volume > 9%/<30%, n = 5), grey weak responder (relative tumor volume > 30%/<78%, n = 4) and black non-responder (relative tumor volume > 78%, n = 4). The dark red line indicates LADs (lamin-associated domains) that are associated in location with LHBs.


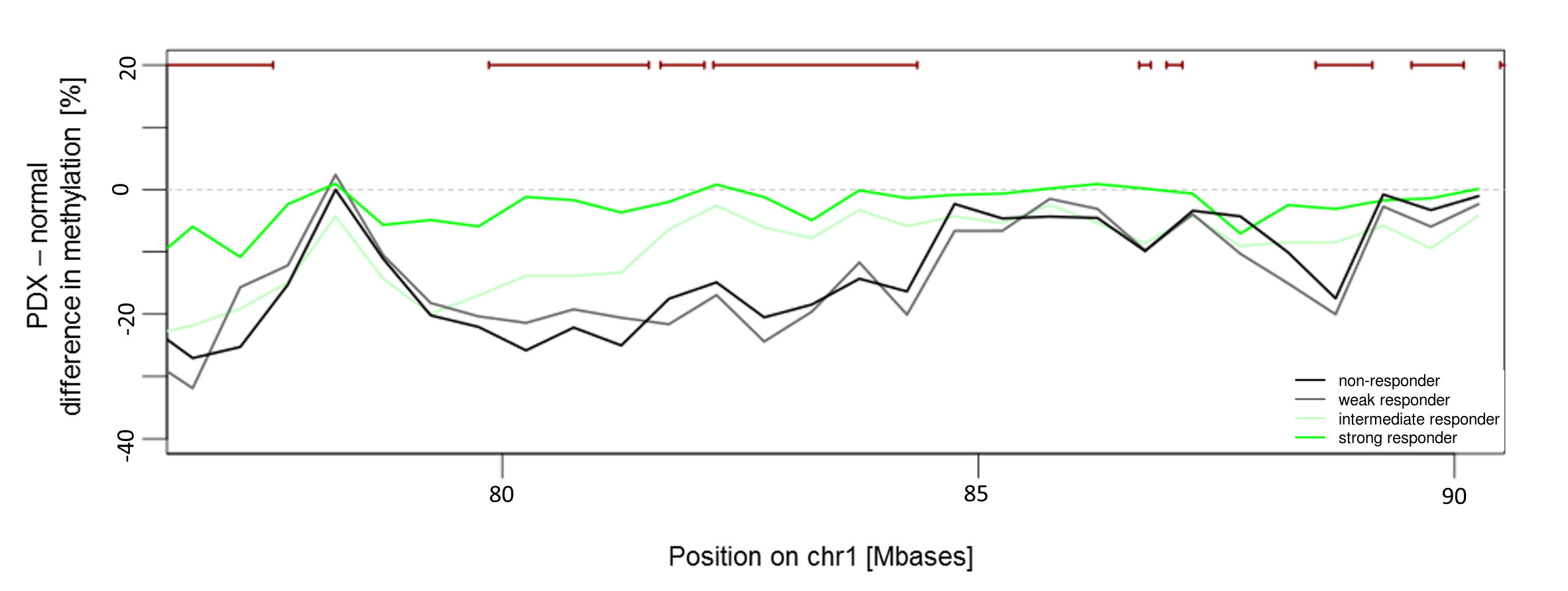


**
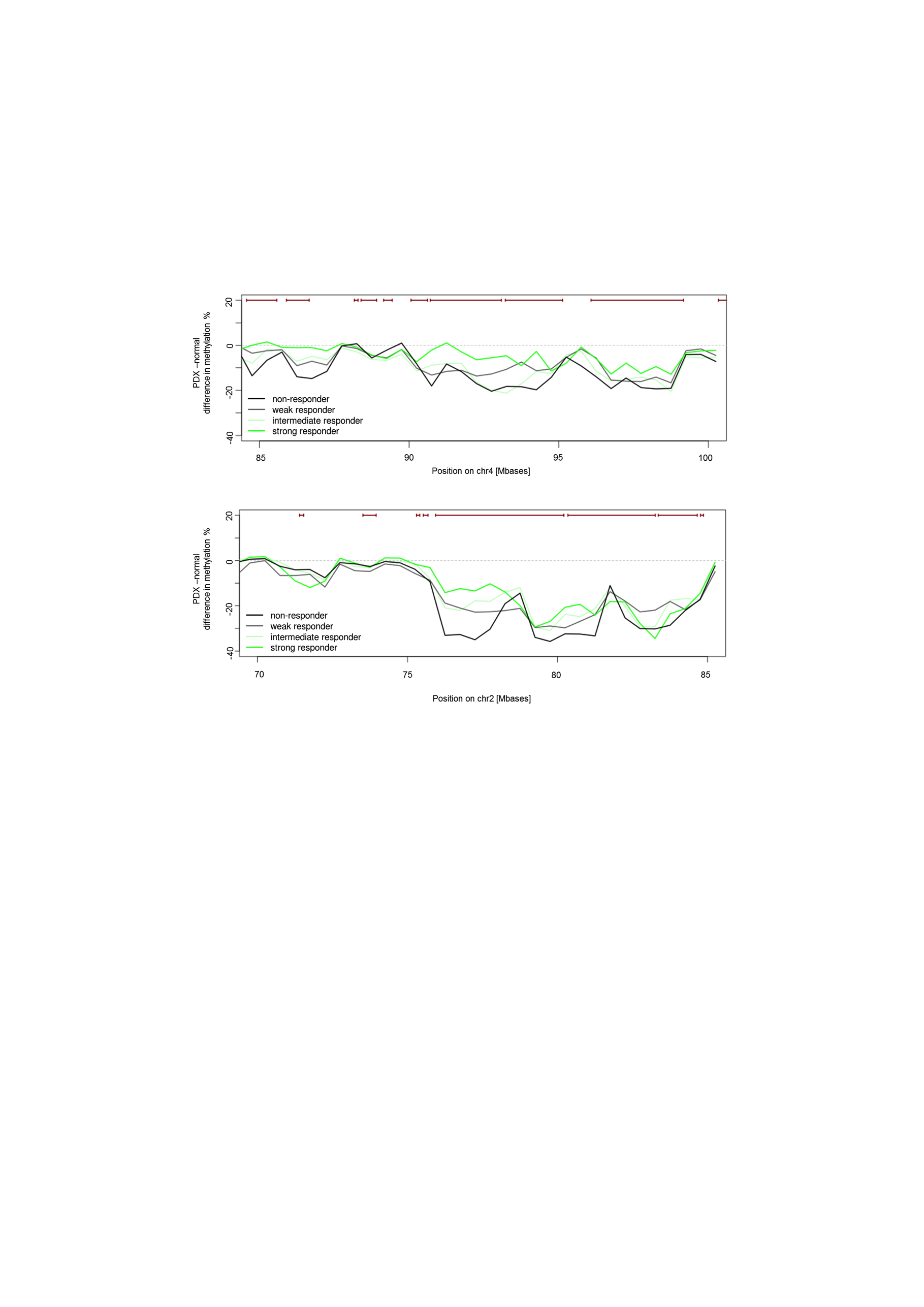
**

**Table S6: Histopathologic evaluation of primary tumor and PDX tumor tissues.**

**Each primary tumor and its corresponding xenograft were evaluated for expression of typical markers for either squamous cell lung cancer (p40), lung adenocarcinoma (TTF-1), or small cell lung cancer (CD56). Individual cases that were negative for all three markers, were further characterized using Cytokeratin markers including CK5, AE1/3, and CK7. The amount of tumor [% ] was determined by evaluating the tumor center using a 200x magnification.**

| **primary tumor** | | **amount of tumor# (%)** | **Xenograft** | **amount of tumor# (%)** | **P40 (nuclear) primary/**  **PDX** | **TTF1 (nuclear)  primary/**  **PDX** | **CD56 (cytoplasm)  primary/**  **PDX** |  | | | | | | | | | | |
| --- | --- | --- | --- | --- | --- | --- | --- | --- | --- | --- | --- | --- | --- | --- | --- | --- | --- | --- |
| 3257 -16 | | 30 | LU7612 | 90 | pos/pos | neg/neg | neg/neg |  | | | | | | | | | | |
| 7657 -13 | | 40 | Lu7766/P8 | 90 | pos/pos | neg/neg | neg/neg |  | | | | | | | | | | |
| 6485 -14+ | | 40 | Lu10005+ | 80 | neg/necrot.  neg. | pos/necrot neg. | neg/necrot.  neg |  | | | | | | | | | | |
| 210 -7 | | 60 | Lu9643 | 90 | pos/pos | neg/neg | neg/neg |  | | | | | | | | | | |
| 541 -14 | | 30 | Lu7913 | 85 | pos/pos | neg/neg | neg/neg |  | | | | | | | | | | |
| 11098 -1 | | 50 | Lu7860 | 90 | pos/pos | neg/neg | neg/neg |  | | | | | | | | | | |
| 10136 -12*** | | 40 | Lu7466*** | 85 | neg/neg | neg/neg | neg/neg |  | | | | | | | | | | |
| 11912 -7 | | 40 | Lu7506 | 80 | pos/pos | neg/neg | neg/neg |  | | | | | | | | | | |
| 9929 -8 | | 30 | Lu7462 | 85 | neg/neg | positive | neg/neg |  | | | | | | | | | | |
| 3971 -4* | | 50 | Lu7298* | 90 | pos/neg | neg/neg | neg/neg |  | | | | | | | | | | |
| 5278 -13 | | 45 | Lu7343 | 90 | pos/pos | neg/neg | neg/neg |  | | | | | | | | | | |
| 5148 -7**** | | 60 | Lu7336/P16**** | 80 | neg/neg | neg/neg | neg/neg |  | | | | | | | | | | |
| 10119 -4 | | 60 | Lu7166 | 90 | pos/pos | neg/neg | neg/neg |  | | | | | | | | | | |
| 10694- 9 | | 70 | Lu7177 | 80 | pos/pos | neg/neg | neg/neg |  | | | | | | | | | | |
|  | + = Diskrepancy probably due to necrosis | | | | | | | | | | | | | | |  | | |
|  | * = Discrepancy between tumor and xenograft | | | | | | | | | | | | | | |  | | |
|  | ** = CK5 and AE1/3 positive, CK7 negative | | | | | | | | | | | | | | |  | | |
|  | *** = CK7 and AE1/3 positive, CK5 negative | | | | | | | | | | | | | | | | | |
|  | **** = CK5 and CK7 positive | | | | | | | |  |  |  | |  | |  | | |  |
|  | # = amount of tumor is judged by evaluating the tumor center using a 200x magnification | | | | | | | | | | |  | |  | | |  | |

**Figure S7: Ingenuity pathway and upstream regulator analyses of the 2,380 genes differentially methylated.**

(**a**) Canonical pathways identified by Ingenuity Pathway Analysis (IPA) associated with genes with significant rDMRs in their promoter/gene body (**b**) and (**c**) visualization of wnt – and DNA methylation.


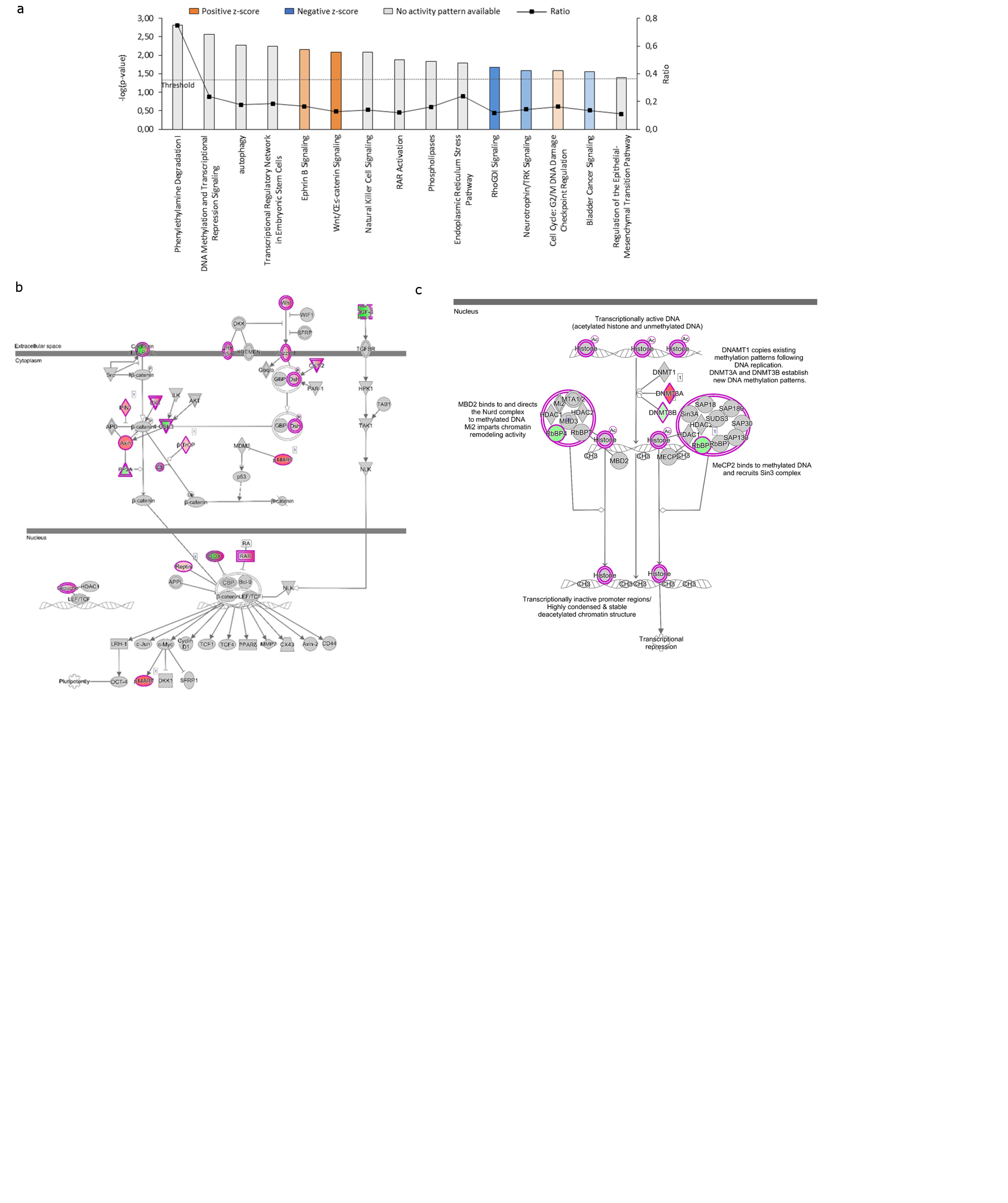


**Figure S8: *LRP12* knock down induces carboplatin resistance.**

**(a)** Validation of the information from Bar et al., 2016 that NCI-H23 cells are sensitive to carboplatin. Identification of a concentration within the linear curve for subsequent experiments. NCI-H23 cells were treated with different carboplatin concentrations without siRNA knock down and cell viability was measured 72h after treatment by neutral red assay. (**b**) Efficiency of siLRP12 knock down measured by quantitative PCR with and without carboplatin treatment (25µg/ml) was examined after 72h. A representative example is shown. (**c**) Cell viability was measured 72h after knock down and treatment with 25µg/ml carboplatin. The used concentration was determined as shown in (a). Three independent experiments were performed, each in triplicates. The mean of the experiments is shown as a line, the triplicate values for the three different experiments are given as circles, squares or triangles, respectively.


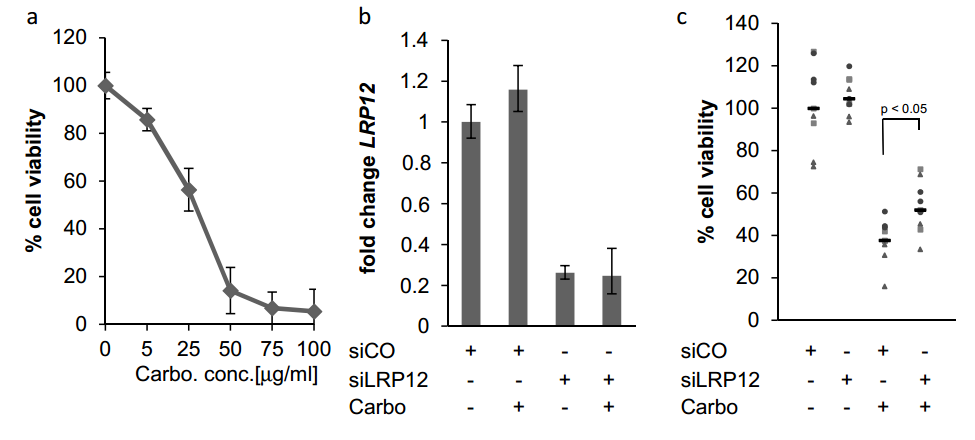


**Table S9: Patient´s data and clinical characteristics of the validation cohort.**

FFPE samples of 35 primary NSCLC samples with 10 pairs of patients with and without relapse (responders/non-responders) of NSCLC and matched for age, sex, histological subtype and tumor stage.

| **Sample** | **Matching pairs** | **Sex** | **UICC stage** | **Histology** | **without relapse** | **with relapse** |
| --- | --- | --- | --- | --- | --- | --- |
| 1 | - | m | IB | ADC | 0 | 1 |
| 2 | 1 | m | IB | ADC | 0 | 1 |
| 3 | 2 | m | IB | others | 0 | 1 |
| 4 | - | m | IIA | ADC | 0 | 1 |
| 5 | 3 | m | IIA | PEC | 0 | 1 |
| 6 | 4 | m | IIB | PEC | 0 | 1 |
| 7 | 5 | f | IIB | ADC | 0 | 1 |
| 8 | - | f | IIB | ADC | 0 | 1 |
| 9 | - | f | IIIA | others | 0 | 1 |
| 10 | 6 | f | IIIA | ADC | 0 | 1 |
| 11 | - | f | IIIA | ADC | 0 | 1 |
| 12 | 7 | m | IIIA | ADC | 0 | 1 |
| 13 | 8 | m | IIIA | ADC | 0 | 1 |
| 14 | 9 | m | IIIA | ADC | 0 | 1 |
| 15 | 10 | m | IIIA | PEC | 0 | 1 |
| 16 | 1 | m | IB | ADC | 1 | 0 |
| 17 | 2 | m | IB | others | 1 | 0 |
| 18 | - | m | IB | ADC | 1 | 0 |
| 19 | - | f | IB | ADC | 1 | 0 |
| 20 | - | m | IIA | PEC | 1 | 0 |
| 21 | 3 | m | IIA | PEC | 1 | 0 |
| 22 | 4 | m | IIB | PEC | 1 | 0 |
| 23 | - | m | IIB | PEC | 1 | 0 |
| 24 | 5 | f | IIB | ADC | 1 | 0 |
| 25 | - | f | IIB | ADC | 1 | 0 |
| 26 | - | f | IIIA | others | 1 | 0 |
| 27 | 6 | f | IIIA | ADC | 1 | 0 |
| 28 | - | f | IIIA | ADC | 1 | 0 |
| 29 | - | m | IIIA | others | 1 | 0 |
| 30 | 7 | m | IIIA | ADC | 1 | 0 |
| 31 | - | m | IIIA | ADC | 1 | 0 |
| 32 | - | m | IIIA | ADC | 1 | 0 |
| 33 | 8 | m | IIIA | ADC | 1 | 0 |
| 34 | 9 | m | IIIA | ADC | 1 | 0 |
| 35 | 10 | m | IIIA | PEC | 1 | 0 |

ADC, adenocarcinoma, PEC, perivascular epithelioid cell tumor, PFS, progression-free survival, OS, overall survival

**Figure S9: *LRP12* DNA hypermethylation as independent predictive factor for clinical outcome in NSCLC.**

**(a)** ROC analyses for *LRP12* methylation, **(b)** *LRP12* methylation level in primary NSCLC tumors with relapse (non-responders) or without relapse (responders). Kaplan-Meier analysis of progression-free survival in 35 NSCLC patients of an independent validation cohort with respect to *LRP12* methylation status (LRP12+ hypermethylated, 16 patients; LRP12-, non-hypermethylated, 19 patients). The statistical significance of the log-rank test is shown. Time to relapse in years is indicated for both groups with individual patients censored. Confidence interval is marked in dark blue.

| **a**  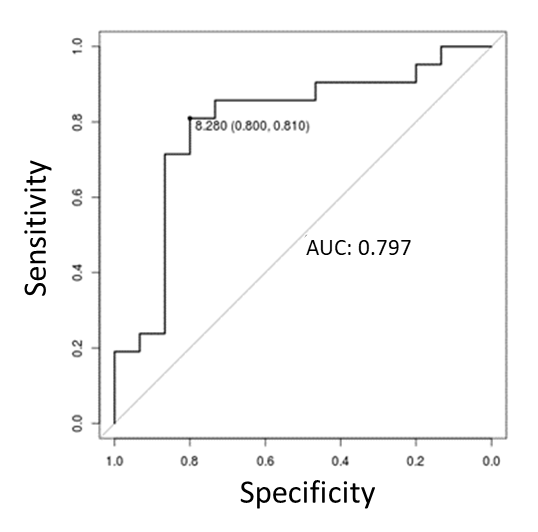 | **b**  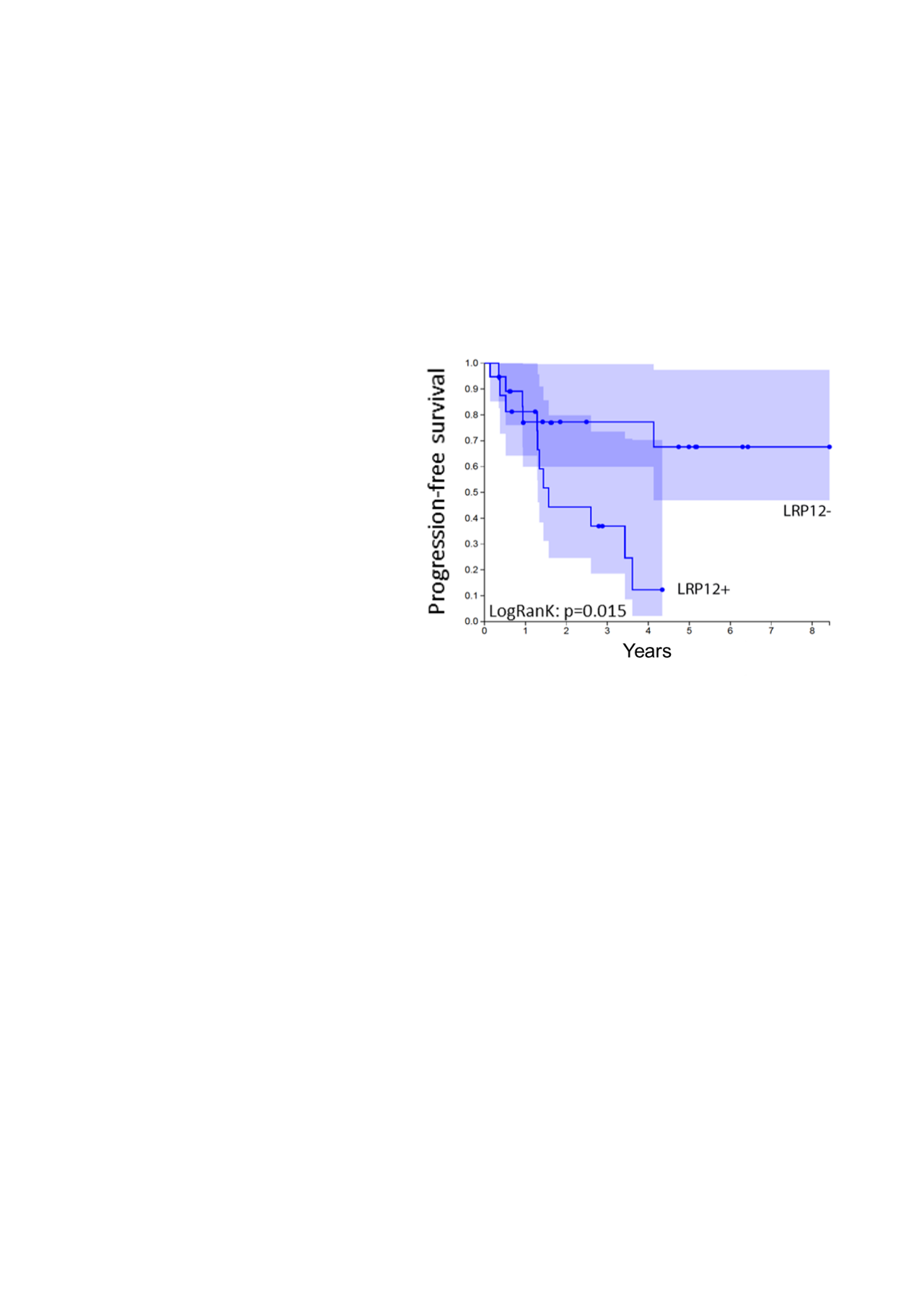 |
| --- | --- |

**Figure S10: *LRP12* DNA hypermethylation as independent predictive factor for clinical outcome in 449 NSCLC patients from the TCGA data set.**

*LRP12* methylation level in primary NSCLC tumors with relapse (non-responders) or without relapse (responders). Kaplan-Meier analysis of progression-free survival in 475 NSCLC patients of an independent validation cohort with respect to *LRP12* methylation status (LRP12+ hypermethylated, 274 patients; LRP12-, non-hypermethylated, 175 patients).


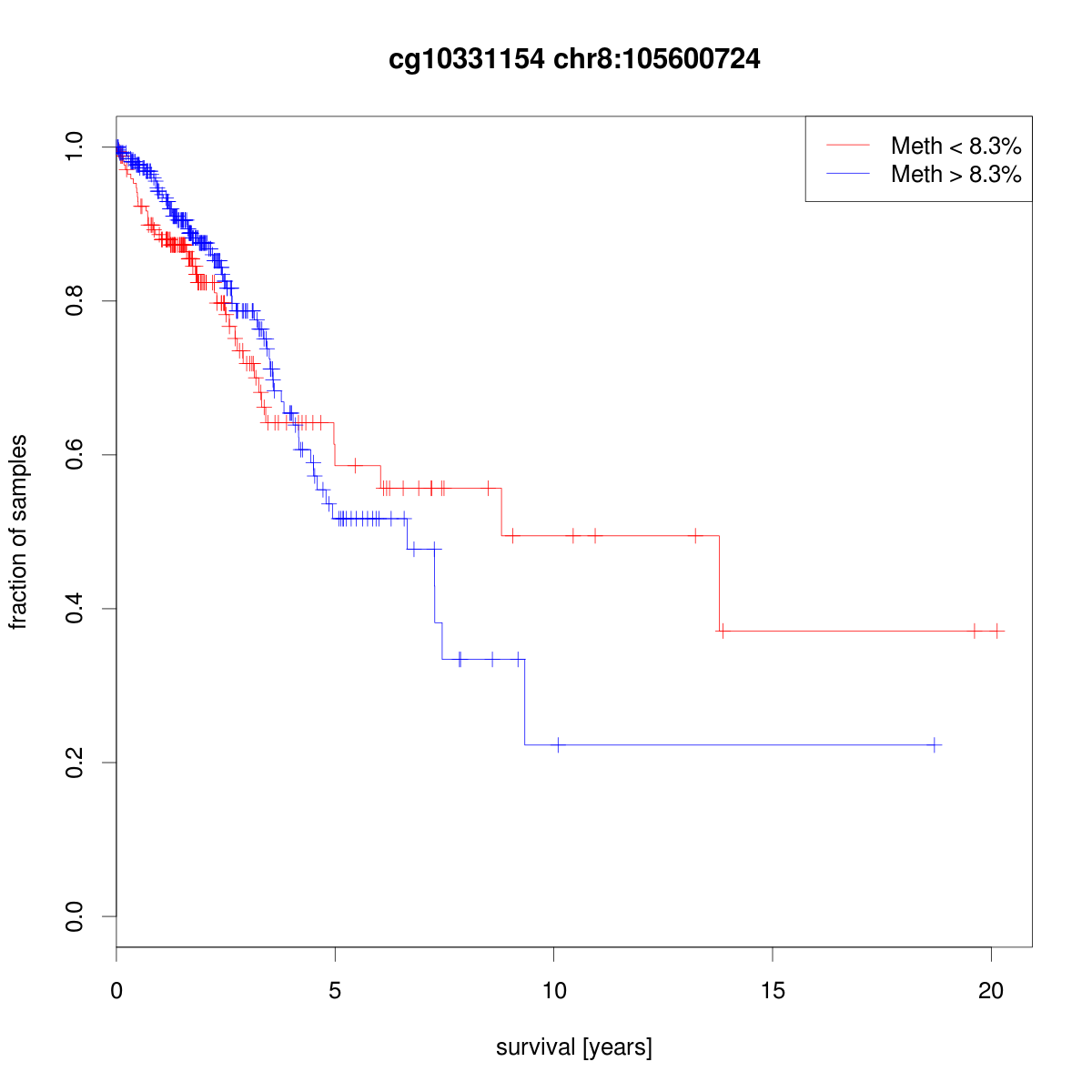


**Additional References**

Bar J, Hasim MS, Baghai T, Niknejad N, Perkins TJ, Stewart DJ, Sekhon HS, Villeneuve PJ, Dimitroulakos J. Induction of Activating Transcription Factor 3 IsAssociated with Cisplatin Responsiveness in Non-Small Cell Lung Carcinoma Cells. Neoplasia. 2016 Sep;18(9):525-35. doi: 10.1016/j.neo.2016.07.004. PubMed PMID:27659012; PubMed Central PMCID: PMC5031866.

Repetto G, del Peso A, Zurita JL. Neutral red uptake assay for the estimation of cell viability/cytotoxicity. Nat Protoc. 2008;3(7):1125-31. doi:10.1038/nprot.2008.75. PubMed PMID: 18600217.
